# Bicarbonate insertion triggered self-assembly of chiral octa-gold nanoclusters into helical superstructures in the crystalline state†

**DOI:** 10.1039/d2sc03463h

**Published:** 2022-08-15

**Authors:** Wei-Dan Si, Kai Sheng, Chengkai Zhang, Zhi Wang, Shan-Shan Zhang, Jian-Min Dou, Lei Feng, Zhi-Yong Gao, Chen-Ho Tung, Di Sun

**Affiliations:** Key Lab of Colloid and Interface Chemistry, Ministry of Education, School of Chemistry and Chemical Engineering, Shandong University Jinan 250100 P. R. China dsun@sdu.edu.cn; School of Aeronautics, Shandong Jiaotong University Ji'nan 250037 People's Republic of China; Shandong Provincial Key Laboratory of Chemical Energy Storage and Novel Cell Technology, School of Chemistry and Chemical Engineering, Liaocheng University Liaocheng 252000 People's Republic of China; School of Chemistry and Chemical Engineering, Henan Normal University Xinxiang 453007 Henan People's Republic of China

## Abstract

Constructing atomically precise helical superstructures of high order is an extensively pursued subject for unique aesthetic features and underlying applications. However, the construction of cluster-based helixes of well-defined architectures comes with a huge challenge owing to their intrinsic complexity in geometric structures and synthetic processes. Herein, we report a pair of unique *P-* and *M-*single stranded helical superstructures spontaneously assembled from *R*- and *S*-Au8c individual nanoclusters, respectively, upon selecting chiral BINAP (2,2′-bis(diphenylphosphino)-1,1′-binaphthalene) and hydrophilic *o*-H_2_MBA (*o*-mercaptobenzoic acid) as protective ligands to induce chirality and facilitate the formation of helixes. Structural analysis reveals that the chirality of the Au8c individual nanoclusters is derived from the homochiral ligands and the inherently chiral Au_8_ metallic kernel, which was further corroborated by experimental and computational investigations. More importantly, driven by the O–H⋯O interactions between (HCO_3_^−^)_2_ dimers and achiral *o*-HMBA^−^ ligands, *R*/*S*-Au8c individual nanoclusters can assemble into helical superstructures in a highly ordered crystal packing. Electrospray ionization (ESI) and collision-induced dissociation (CID) mass spectrometry of Au8c confirm the hydrogen-bonded dimer of Au8c individual nanoclusters in solution, illustrating that the insertion of (HCO_3_^−^)_2_ dimers plays a crucial role in the assembly of helical superstructures in the crystalline state. This work not only demonstrates an effective strategy to construct cluster-based helical superstructures at the atomic level, but also provides visual and reliable experimental evidence for understanding the formation mechanism of helical superstructures.

## Abstract 2

A pair of unprecedented helical superstructures *via* self-assembly of inherently homochiral Au_8_ nanoclusters, [Au_8_(*R*/*S*-BINAP)_3_(*o*-HMBA)_2_]·2(HCO_3_), is obtained in the crystalline state, in which the HCO_3_^−^ ions act as the bridge.

## Graphical abstract

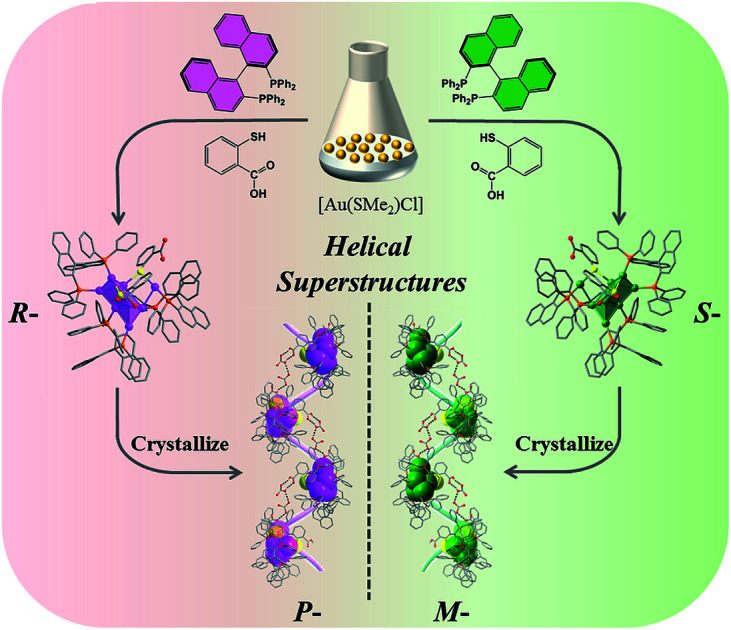

## Introduction

Chirality appears to be widespread in nature and occurs on various length scales ranging from the subatomic to molecular, to supramolecular level, and even to galaxies.^1–3^ In contrast to molecular chirality that essentially refers to point, axis and plane chirality, supramolecular chirality is represented by the periodic asymmetric packing of chiral or achiral molecules, which is ubiquitous in biological systems.^4–7^ A typical example of supramolecular chirality is the secondary structural proteins which display miscellaneous architectures, such as the α-helix, β-sheet, and coiled-coil helix bundle. Within the subset of chiral superstructures, the helix is a typical topological structure that can either be left-handed (*P*) or right-handed (*M*) depending on the screwing motion directions.^8,9^ In recent years, helical superstructures have received considerable attention due to their fascinating geometric features and promising applications in the fields of chiral recognition, sensors and asymmetric catalysis.^10–12^ However, how to synthesize helical superstructures with precise size, structure and composition is still a problem to be solved.

Supramolecular interactions (non-covalent interactions), such as hydrogen bonding, π⋯π stacking, C–H⋯π, and electrostatic interaction, are the driving forces for constructing and crystallizing aesthetical supramolecular architectures with high regularity at the atomic level.^13–16^ Through these supramolecular interactions, many supramolecular architectures based on coinage metal nanoclusters of precise structures and compositions have been successfully constructed.^17–22^ As we know, some supramolecular helical structures related to hydrogen bond interactions can be established *via* introducing carboxylic acids. However, to date, cluster-based helical architectures have been rarely reported due to the lack of structural stability and difficulty in atomic-level characterization.^23–27^ Thus, in-depth investigation of helical superstructures of ordered arrangement units at the atomic level is still in the hysteretic state.

Protective ligands on the surface of coinage metal nanoclusters are particularly crucial for dominating their sizes, structural patterns and physicochemical properties; moreover, appropriate choice of surface ligands is directly correlated to their packing in the crystalline state,^28–34^ which may result in diverse superstructures. Since the observation of circular dichroism (CD) signals in ligand-protected gold nanoclusters by Schaff and Whetten in 1998,^35^ a series of chiral organic ligands have been extensively employed to functionalize coinage metal nanoclusters and significant progress has been achieved in recent years.^36–44^ Besides, it is found that protective ligands containing –COOH groups are of potential interest for constructing various cluster-based supramolecular architectures *via* intermolecular interactions. For example, both the structurally determined Ag_44_ and Au_102_ nanoclusters protected by the *p*-mercaptobenzoic acid (*p*-H_2_MBA) ligands displayed well-organized arrangements in their crystal lattices driven by hydrogen bonding.^17,45–47^ Thus, the selection of suitable surface ligands determines not only the structures and properties of the metal nanoclusters but also the superstructures formed upon entering condensed states.

Here, we exquisitely select chiral 2,2′-bis(diphenylphosphino)-1,1′-binaphthalene (BINAP) as the major protective ligand to endow gold nanoclusters with chirality, and *o*-mercaptobenzoic acid (*o-*H_2_MBA) carrying –COOH groups as the auxiliary ligand to crystallize diverse gold cluster-based superstructures. A pair of enantiomers [Au_8_(*R*/*S*-BINAP)_3_(*o*-HMBA)_2_]·2HCO_3_ (*R*/*S*-Au8c) in which two HCO_3_^−^ anions were formed *in situ* by capturing the atmospheric CO_2_ has been successfully synthesized. Strikingly, *R*-Au8c and *S*-Au8c demonstrate *P-* and *M-*helical superstructures running along the 2-fold screw axis in the crystalline state, respectively, driven by the synergistic effect of intermolecular hydrogen bonds and electrostatic interactions. Their chirality was elucidated *via* X-ray crystallography, CD spectra and theoretical calculations, which indicated that the chirality arose from the metallic kernel and ligand shell. It was also found that Au8c exhibits crystallization-induced emission enhancement (CIEE).^48^ This study provides an excellent structural model to understand the self-assembly of cluster-based supramolecular helixes at the atomic level and inspires us to construct helical superstructures in a controllable manner.

## Results and discussion

### Synthesis discussion

**Scheme 1:**
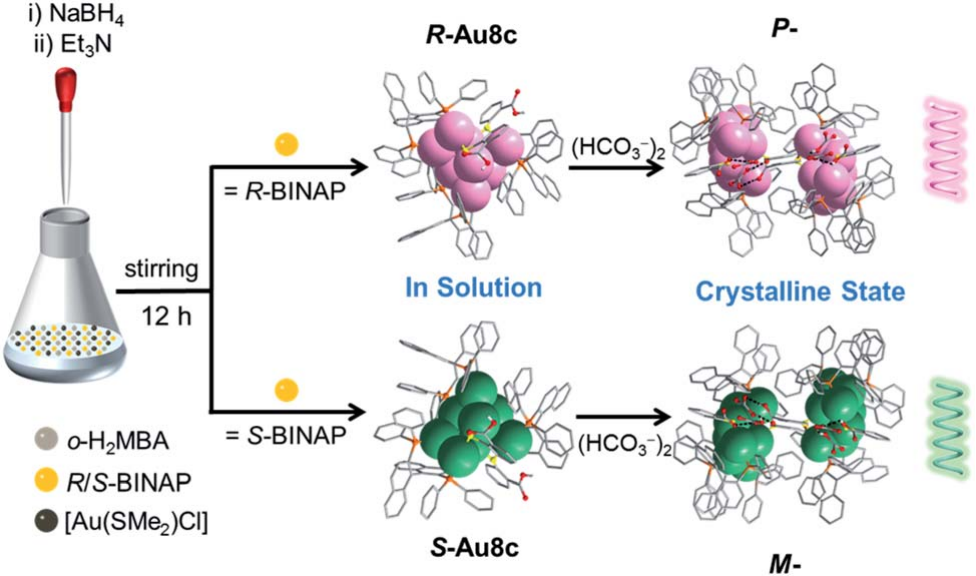
Synthetic routes to *R*/*S*-Au8c in solution and the homochiral *P-*/*M*-helixes formed in the crystalline state.

In a representative reaction, a mixture of [Au(SMe_2_)Cl], *R*- or *S*-BINAP and *o-*H_2_MBA was dissolved in a mixed solvent of CH_3_OH and CH_2_Cl_2_. A freshly prepared aqueous solution of NaBH_4_ was employed to initiate the reduction process, followed by the addition of Et_3_N at room temperature (Scheme 1). After two weeks, plate-like orange crystals of Au8c were obtained by layering *n*-hexane on the dichloromethane solutions of the corresponding gold nanoclusters, with a yield of ∼15% based on [Au(SMe_2_)Cl] (Fig. S1†). Single-crystal X-ray diffraction (SCXRD) analysis revealed that the unit cell of Au8c contains two unexpected HCO_3_^−^ anions, which were connected together as a (HCO_3_^−^)_2_ dimer by hydrogen bonds, without an additional source in the reaction. Under alkaline conditions, such HCO_3_^−^ or carbonates (CO_3_^2−^) were occasionally observed in coinage metal nanoclusters and they are usually derived from the conversion of atmospheric CO_2_.^49–51^ Thus we speculated that the HCO_3_^−^ anions were generated *in situ* by capturing atmospheric CO_2_ in the presence of Et_3_N during the reaction process (CO_2_ + H_2_O + Et_3_N → Et_3_NH^+^ + HCO_3_^−^).^52^

To prove our hypothesis, we performed a controlled experiment under a CO_2_ atmosphere represented by *R*-Au8c. As speculated, the yield (∼25%) of *R*-Au8c nanoclusters was slightly increased. For comparison, we also performed an experiment under a N_2_ atmosphere, and another new nanocluster, [Au_8_(*R*-BINAP)_3_(*o*-MBA)_2_] (*R*-Au8d), was isolated without any trapped HCO_3_^−^ anions (Fig. S2 and Table S1†). Furthermore, different amounts of NaHCO_3_/KHCO_3_ ranging from 0.005 mmol to 0.03 mmol were directly added to the reaction solution to improve the yield of *R*-Au8c, but no crystalline products were obtained. Based on the above observation, intentionally added HCO_3_^−^ may disrupt the acid–base equilibrium of the reaction system (OH^−^ + HCO_3_^−^ ⇋ H_2_O + CO_3_^2−^), which was unfavourable for the crystallization of *R*-Au8c.

Moreover, we also tried to replace *o-*H_2_MBA with *m-*H_2_MBA and *p-*H_2_MBA ligands to explore the effect of positional isomers on the helical arrangement of Au_8_ nanoclusters. When *o-*H_2_MBA was replaced by *m-*H_2_MBA, [Au_8_(*R*-BINAP)_3_(*m*-MBA)_2_] (*R*-Au8e) was obtained (Fig. S3 and Table S1†); whereas when *o-*H_2_MBA was replaced by *p-*H_2_MBA, no crystalline products were obtained. These results suggested that even a subtle variation of positional isomeric ligands (*o*/*m*/*p-*H_2_MBA) can make a dramatic effect on the formation and helical arrangement of nanoclusters (for some detailed discussions please see the below section). The solid-state Fourier transform infrared (FT-IR) spectrum further confirmed the presence of HCO_3_^−^ anions in Au8c, and it was comparable to that of NaHCO_3_ (Fig. S4†) and similar to that in the literature.^53^ Phase purity of *R-*/*S-*Au8c was characterized by powder X-ray diffraction (PXRD) (Fig. S5†). More details of synthesis and characterization are shown in the ESI.†

### Crystal structures of *R*/*S*-Au8c

**Fig. 1:**
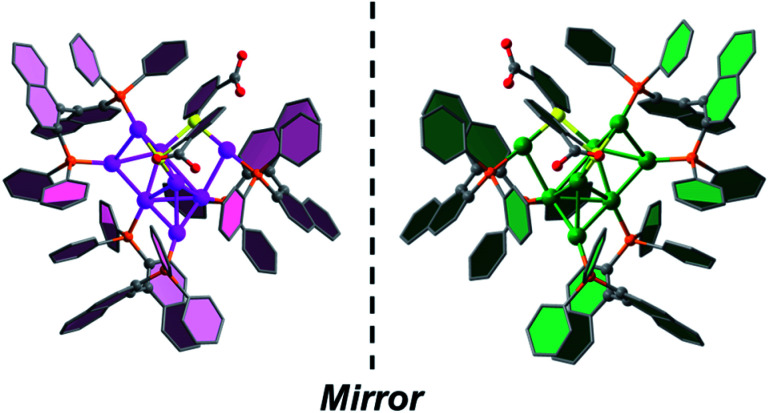
Single-crystal structures of dicationic *R*-Au8c and *S*-Au8c nanoclusters. Color labels: pink and green, Au; yellow, S; orange, P; gray, C; red, O. All hydrogen atoms are omitted for clarity.

SCXRD analysis revealed that the basic unit of *R*/*S*-Au8c contains one [Au_8_(*R*/*S*-BINAP)_3_(*o-*HMBA)_2_]^2+^ nanocluster and two HCO_3_^−^ anions. Both *R-*Au8c and *S*-Au8c crystallize in the orthorhombic chiral space group *P*2_1_2_1_2_1_, with Flack parameters of 0.012(12) and 0.041(8), respectively, indicating their absolutely chiral configurations (Table S1†). To better understand their geometric structures, the dicationic *R*/*S*-Au8c enantiomers without anions are depicted in Fig. 1. The Au8c individual nanocluster has an Au_7_ core, which can be regarded as the fusion of an Au_5_ trigonal bipyramid and an Au_5_ square pyramid through sharing a triangular Au_3_ face, where the Au_7_ core is attached by an exterior gold atom in *R*-Au8c and *S*-Au8c, respectively (Fig. S6†). Such an additional gold atom at the asymmetric *exo* position lowers the symmetry of Au8c, leading to the *C*_1_ symmetry and perfect mirror symmetry of the Au_8_ metallic kernels. Thus, Au8c demonstrates an intrinsically chiral kernel, which is not usual in chiral gold nanoclusters.^36,41,42^ The average Au–Au bond length of *R*-Au8c is 2.83 Å (range: 2.65–3.21 Å), which is slightly shorter than the 2.88 Å in bulk gold, indicating the existence of aurophilic interactions.^54^

Compared to the lowest-energy planar Au_8_ isomers derived from DFT calculations,^55^Au8c exhibits a new type of nonplanar configuration that can be ascribed to the surface ligand effects.^30^ On the periphery of Au8c, there are totally three μ_2_-bridging chiral BINAP and two μ_2_-bridging *o-*HMBA^−^ ligands. Each BINAP ligand has a flexible conformation due to the rotation of the C–C single bond, which is unconducive to the planar geometry of Au_8_; all of them ride on the edges of the Au_8_ metallic kernel to form contorted Au_2_P_2_C_4_ eight-membered rings (average Au–P: 2.29 Å in *R*-Au8c). One of the two *o-*HMBA^−^ ligands bridge the exterior gold atom (Au1) and one Au atom (Au6) at the vertex of the shared Au_3_ triangle, and the other bridges a base edge (Au4–Au7) of the Au_5_ square pyramid by a μ_2_-S atom (Fig. S6†). The bond angles of Au1–S1–Au4 and Au7–S2–Au8 in *R*-Au8c are 97.3(2)° and 92.1(3)° (Table S2†), respectively, which are smaller than the typical Au–S–Au average bond angle in thiolate-protected gold nanoclusters.^56,57^

**Fig. 2:**
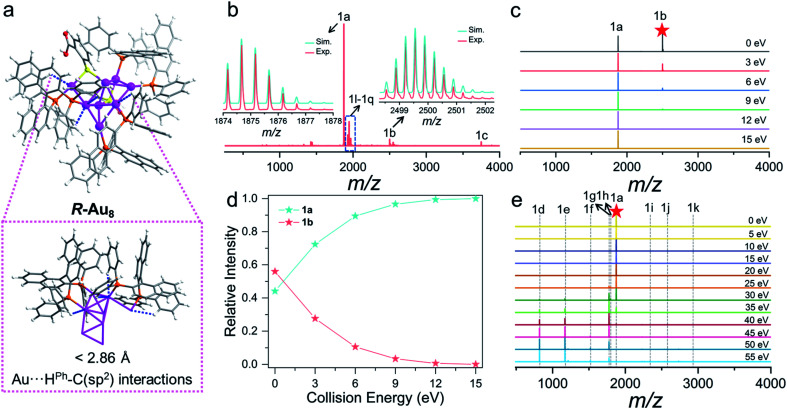
(a) Optimized structure of *R*-Au8c showing Au⋯H–C interactions. (b) Positive-ion mode ESI-MS of the Au8c dissolved in CH_3_OH. Inset: the experimental (red trace) and simulated (cyan trace) isotopic patterns (peak 1a and 1b). (c) CID mass spectra of 1b measured at the collision energy of 0–15 eV. (d) Relative intensity of the resulting 1b fragment ions collected at the collision energy of 0–15 eV. (e) CID mass spectra of 1a measured at the collision energy of 0–55 eV.

Unexpected Au⋯H–C electrostatic interactions between the gold framework and the chiral BINAP in *R*/*S*-Au8c, which are a new kind of hydrogen bond according to IUPAC definitions in 2011,^58^ are highlighted. In terms of the axially chiral BINAP ligands, two biaryl backbones can agilely rotate around the axis to form a dihedral angle, further making the aromatic rings inclined to establish Au⋯H–C interactions. In the crystal structures of *R*-Au8c and *S*-Au8c, the hydrogen atoms from the phenyl rings of BINAP are close to the coordinated gold atoms. Accordingly, their optimized structures also display some short Au⋯H distances, shorter than the sum of their van der Waals radii of 2.86 Å,^59^ and the corresponding Au⋯H–C angles are about 120° (Fig. 2a and S7†). In comparison with the [Au_6_]^2+^ nanoclusters reported by Konishi,^60^ the Au⋯H–C interactions are relatively weak, which may be due to the smaller Au⋯H–C angles in *R*/*S*-Au8c. Taking *R*-Au8c as an example, noncovalent interaction analysis was conducted using Multiwfn 3.7 (dev) to investigate the Au⋯H–C interactions (Fig. S8†).^61^ Benefiting from these intramolecular Au⋯H–C interactions, the metallic kernels of two Au8c enantiomers can be “locked in” to some extent and can facilitate the enantioselectivity. The chiral metal kernels give feedback to the surficial *o-*HMBA^−^ ligands through coordinated bonds, which boosts “chiral transfer” between BINAP and *o-*HMBA^−^.

### ESI and CID mass spectrometry of Au8c

Positive-ion mode ESI-MS of Au8c dissolved in CH_3_OH was conducted to confirm the composition and charge state (Fig. 2b). It displayed a series of peaks corresponding to multi-charge state species from +1 to +3 in the *m*/*z* range of 500 to 5000 (Fig. 2a, S9 and Table S3†). The most dominant peak 1a at *m*/*z* 1874.68 can be assigned to the molecular ion peak [Au_8_(BINAP)_3_(*o-*HMBA)_2_]^2+^ (calc. 1874.67). Therefore, the metallic kernels of Au_8_ nanoclusters carry four free electrons and exhibit a typical prolate shape based on superatom electronic theory.^62,63^ There is +1 charge species 1c centered at *m*/*z* 3748.34, corresponding to [Au_8_(BINAP)_3_(*o-*HMBA)_1_(*o-*MBA)_1_]^+^ (calc. 3748.32), resulting from the deprotonation of one *o-*HMBA^−^ in species 1a. Besides, the *o-*HMBA^−^ on the metallic surface with the uncoordinated –COOH group can provide a platform to construct dimers, trimers, and polymers with the bridge of hydrogen bonds.^13,64^ As shown in Fig. 2b, we observed +3 charged novel species at *m*/*z* 2499.56 (1b, calc. 2499.55) which can be identified as an Au8c dimer possibly bonded by intermolecular hydrogen bonds.

To prove that the above-mentioned hydrogen-bonded dimer indeed exists in solution, we trapped this species 1b for further CID mass spectrum analysis with N_2_ gas in the collision energy of 0–15 eV (Fig. 2c), given that CID is a versatile technique to qualitatively probe hydrogen bonds.^65^ Notably, even when the collision energy is 0 eV, 1b can spontaneously dissociate into 1a (Fig. 2c and d), revealing the breakage of very weak bond strength. Considering the crystallographic feature of Au8c, we can qualitatively infer that 1b is a hydrogen-bonded dimer. The relative intensity of monomeric Au8c*vs.* dimeric Au8c is dynamically changeable and highly dependent on the collision energy. Upon increasing the collision energy from 0 eV to 15 eV, the 1b peak progressively faded away, while the 1a peak maintained a continuous rise to be the main species. In brief, such a CID collision-energy-dependent depolymerization of the Au8c dimer indicates the existence and stability of hydrogen-bonded dimer species in solution,^65^ conducive to uncovering the probable assembly mechanism toward helical superstructures (see below).

In addition, to understand bond fission in monomeric Au8c, we also targeted the molecular ion peak 1a to perform CID experiments. By plotting the relative intensity of dominant fragments as a function of collision energy (Fig. 2e), it was noticed that the relative intensity of target 1a was almost unchanged when the collision energy was less than 15 eV and it began to decrease sharply when the collision energy continued to increase, which indicates that 1a is quite stable in low-energy CID processes. Upon increasing the collision energy, as shown in Fig. 2e, a series of novel fragment peaks 1d–1k sprang up, presenting various relative intensities. Intriguingly, all larger species 1h–1k possess four valence electrons as 1a, implying their higher stability.^62^ Correlating the total CID mass spectra of 1a to the relative intensity distribution of the varisized fragments (Fig. S10a†), three possible types of gas-phase dissociation patterns, such as ligand fragmentation, core fission, and ligand loss, were summarized (Fig. S10b†).^66,67^ Each pattern competed with each other to be dominant in the collision dissociation process. Before 40 eV the propensity of 1a is to undergo ligand fragmentation *via* C–H bond activation to generate 1g along with the loss of neutral AuH.^68,69^ Then core fission would be the primary dissociation pathways of 1a, displayed as the following equation: [Au_8_(BINAP)_3_(*o-*HMBA)_2_]^2+^ → [Au_*n*_(BINAP)_1_(*o-*HMBA)_*m*_]^+^ + [Au_8−*n*_(BINAP)_2_(*o-*HMBA)_2−*m*_]^+^ (*n* = 1, 2, 3, *m* = *n* − 1); the relative intensities of smaller fragments [Au_*n*_(BINAP)_1_(*o-*HMBA)_*m*_]^+^ (1d, 1e, 1f) far exceed those of larger fragments [Au_8−*n*_(BINAP)_2_(*o-*HMBA)_2−*m*_]^+^ (1i, 1j, 1k). In terms of core fission patterns, when *n* = 1 or 2, the corresponding dissociation pathway may be preponderant because the relative intensity of [Au_*n*_(BINAP)_1_(*o-*HMBA)_*m*_]^+^ is higher than that when *n* = 3.^70^ In addition, 1a can also dissociate an *o-*HMBA^−^ ligand *via* Au–S bond breaking, and lose a hydrogen proton to generate species 1h ([1a–*o-*HMBA^−^–H^+^]^2+^). More details are provided in the ESI (Fig. S10, S11 and Table S4†).

### UV-vis spectra and electronic structure of Au8c

**Fig. 3:**
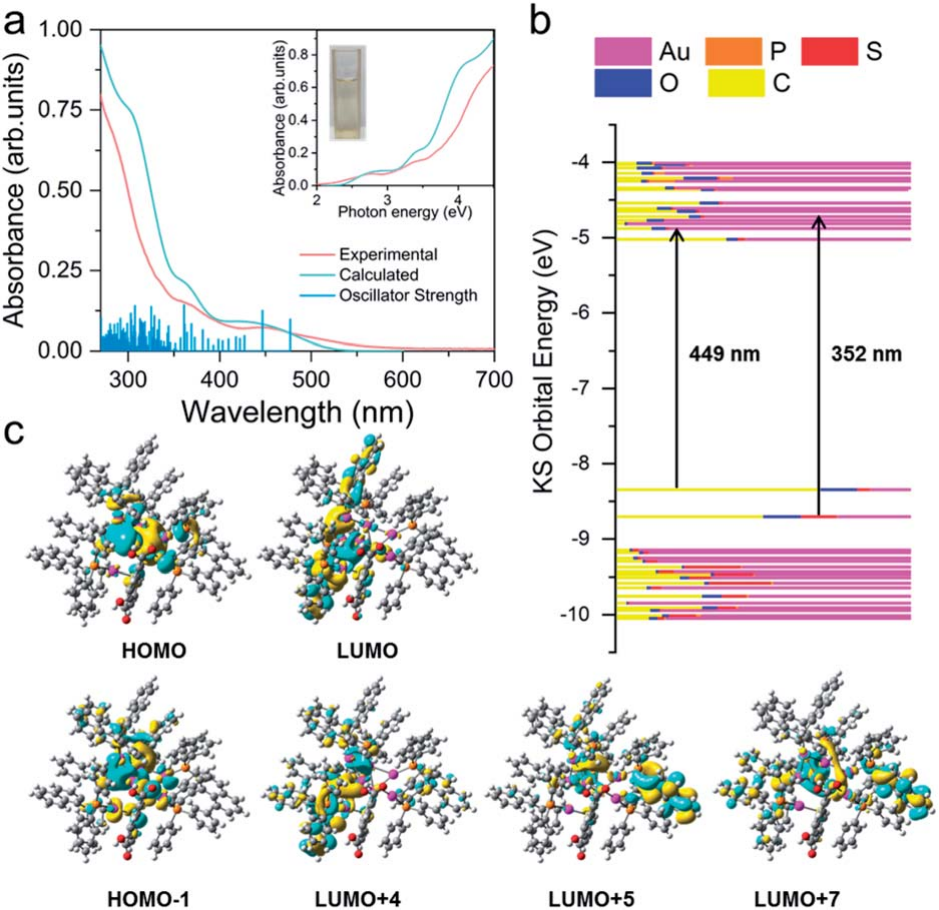
(a) Experimental (pink line) and theoretical (cyan line) UV-vis absorption spectra of Au8c. Inset: experimental (pink line) and theoretical (cyan line) photon-energy plots of Au8c. (b) Kohn–Sham molecular orbital energy level diagram and the associated populations of atomic orbitals in each KS molecular orbital for *R*-Au8c. (c) The calculated HOMO, HOMO−1, LUMO, LUMO+4, LUMO+5 and LUMO+7 orbitals of *R*-Au8c.

To explore the relationship between the crystal structures and optical absorption properties of Au8c nanoclusters, we subsequently carried out time-dependent density functional theory (TD-DFT) calculations. The UV-vis absorption spectrum of Au8c was measured in a dilute solution of CH_3_OH at room temperature. No decomposition was observed in CH_3_OH stored for at least one week (Fig. S12†), suggesting good stability of individual Au8c in solution. The experimental UV-vis absorption spectrum is well reproduced in the calculated one with regard to the spectral profiles (Fig. 3), which can be attributed to the stable structure of individual clusters and the suitable exchange-correlation functional. As shown in Fig. 3, the experimental absorption peak at 2.76 eV (449 nm) in the low-energy region corresponds to an adjacent calculated band, which is mainly composed of two calculated excitations at 2.78 eV (446 nm) and 2.60 eV (477 nm), with strong oscillator strength values of 0.0416 and 0.0326, respectively. Molecular orbital analysis suggested that the most likely transitions are the electronic transitions between the HOMO, which is mainly localized on the 5d and 6s atomic orbitals of Au atoms, and several energetically close lying LUMOs (LUMO+1/LUMO+4/LUMO+5/LUMO+7), involved in the metal-to-ligand charge transfer (MLCT) and metal-to-metal charge transfer (MMCT) (6s/5d → 5p). In the high-energy region, there are two experimental bands centered at 3.52 eV (352 nm) and 4.22 eV (294 nm), corresponding to the calculated absorption bands centered at 3.43 eV (361 nm) and 4.03 eV (307 nm), which have oscillator strength values of 0.0475 and 0.0467, respectively. The absorption band of 3.43 eV primarily originates from HOMO−1 → LUMO+4/LUMO+6 and HOMO → LUMO+9 transitions, which mainly comprise MLCT and a small amount of ligand-to-ligand charge transfer (LLCT) from the p orbitals of S atoms in *o-*HMBA^−^ to the π* orbitals of BINAP ligands. As for the calculated absorption band at 4.03 eV (307 nm), the transitions are more complicated because multiple orbitals are involved. More calculated details of Au8c are listed in Fig. S13 and Tables S5–S7.† Noteworthily, the diverse electronic transitions existing in Au8c provide essential information for us to understand the electronic structure of Au8c as well as for the subsequent investigations on the origin of chirality.

### Chirality of *R*/*S*-Au8c

**Fig. 4:**
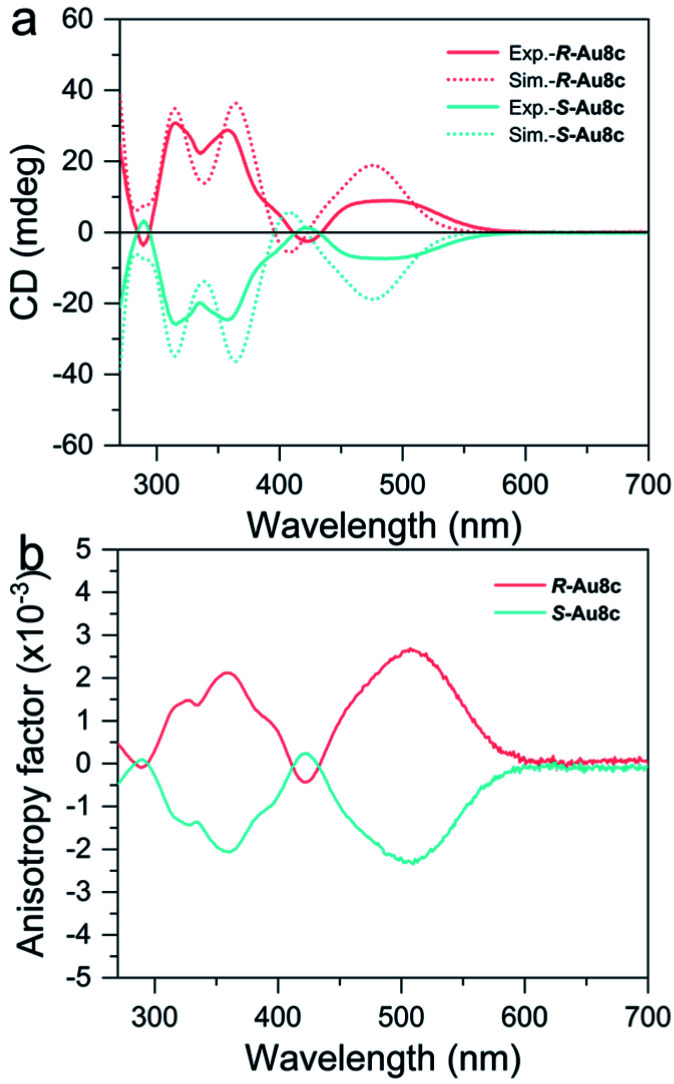
(a) The CD spectra of *R*-Au8c (pink trace) and *S*-Au8c (cyan trace) dissolved in CH_3_OH. (b) Corresponding anisotropy factor of *R*-Au8c (pink trace) and *S*-Au8c (cyan trace). Dot lines: simulated CD spectra.

Next, we measured the CD spectra of *R*-Au8c and *S*-Au8c in a diluted CH_3_OH solution. As shown in Fig. 4, the calculated and experimental CD spectra of Au8c enantiomers both show perfect mirror image chiroptical responses. Taking *R*-Au8c as a representative, the experimental CD spectrum has two sharp signal peaks at 315 and 358 nm, and a broad signal peak approximately at 486 nm, which are in good agreement with the calculated ones. The experimental broad CD peak of *R*-Au8c at the low energy gives the maximum anisotropy factor *g*_abs_ value reaching 2.5 × 10^−3^, which is higher than that in our previous report on BINAP-protected Au_19_ and Au_11_ nanoclusters,^71^ whose chiroptical activities originate from the distorted core and chiral ligands. Such a CD peak corresponds to the experimental absorption peak at 449 nm, and it is related to the MLCT transition as well as the MMCT transition of the inherently chiral metal core. This suggests that the inherently chiral Au_8_ kernel plays a crucial role in enhancing the *g*_abs_ values. Moreover, two CD signals at 315 and 358 nm in the high-energy band also can match with the absorption peaks at 294 nm and 352 nm, respectively, which mainly arise from the MLCT transition from the Au_8_ kernel to BINAP/*o-*HMBA^−^ ligands together with small LLCT transition involving chiral BINAP to *o-*HMBA^−^. Furthermore, the continuous chirality measure (CCM) values of the metallic kernel and the metallic kernel coordinated by S and P atoms of *R*-Au8c and *S*-Au8c were calculated to account for the chiral origins.^72,73^ The CCM value of the Au_8_ kernels in *R*-Au8c and *S*-Au8c is 12.60, indicating their inherently chiral metal kernel. When the surface of Au_8_ kernels was ligated with S and P atoms from ligands, the CCM values show only a slight increase (Table S8†). These results reveal that the chirality of individual Au8c is not only derived from the ligation of chiral BINAP ligands, but also from the intrinsic chiral metal kernel.

### Crystalline state helical self-assembly of chiral Au8c

**Fig. 5:**
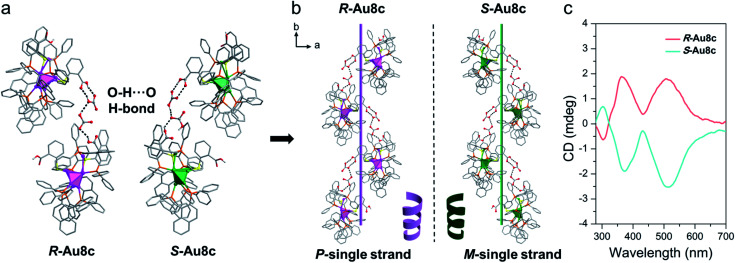
(a) The O–H⋯O hydrogen bonds existing between two adjacent *R*-Au8c and *S*-Au8c involving the (HCO_3_^−^)_2_ dimer. (b) Perspective diagrams of supramolecular chirality for *R*-Au8c and *S*-Au8c indicating the asymmetric packing into *P*- and *M*-single strands, respectively. (c) CD spectra of *R*-Au8c (pink line) and *S*-Au8c (cyan line) in the crystalline state under ambient conditions. Color labels: pink and green, Au; orange, P; yellow, S; gray, C; red, O; white, H.

In nature, typical secondary structures exist in peptides or proteins through helical ordered arrangements of simple primary structures.^74^ Notably, the inserted (HCO_3_^−^)_2_ dimers offer a good bridge to connect adjacent Au_8_ nanoclusters so that individual Au8c enantiomers can transfer their chirality and form helical superstructures during the self-assembly process. Along the *b* axis, neighbouring Au8c nanoclusters are locked together compactly *via* rich O–H⋯O hydrogen bonds supported by the (HCO_3_^−^)_2_ dimers and the –COOH group of *o-*HMBA^−^ ligands on the surface of Au8c, namely O–H⋯O (–COOH → (HCO_3_^−^)_2_ and (HCO_3_^−^)_2_ → −COOH) (Fig. 5a). The average O–H⋯O distance in *R*-Au8c is 2.72 Å (range: 2.65–2.79 Å) with an average O–H⋯O bond angle of 146.88° (range: 126.6–159.8°) (Table S9†). Driven by these O–H⋯O hydrogen bonds, the Au8c enantiomers imitate the behaviour of biomolecules to assemble into helical secondary structures along the 2_1_ screw axis (Fig. 5b). *R*-Au8c and *S*-Au8c exhibit an identical stacking modality, but generate left-handed (*M*-single) and right-handed (*P*-single) helical superstructures, respectively (Fig. 5a). To the best of our knowledge, such helical architectures driven by hydrogen bonded interactions located between gold nanoclusters and inorganic ions are still very rare.

To understand the crucial role exerted by inserted (HCO_3_^−^)_2_ dimers in constructing helical superstructures, a comparative analysis of *R*-Au8d without HCO_3_^−^ was performed. In the same direction (*b* axis), *R*-Au8d also exhibits a helical arrangement running along the 2_1_ screw axis (Fig. S14†). Varying from *R*-Au8c, due to the deprotonation of *o-*H_2_MBA ligands and the absence of HCO_3_^−^ anions, the helical superstructure of *R*-Au8d is stabilized by C–H⋯O (average: 2.588 Å) and C–H⋯π (2.808 Å) interactions (Fig. S14†). Additionally, compared to *R*-Au8c, the stability of the *R*-Au8d helix should be weaker because the O–H⋯O hydrogen bond interactions are generally stronger than C–H⋯O and C–H⋯π interactions.^75^ The above results indicate that the inserted (HCO_3_^−^)_2_ dimers are indispensable for the formation of stable helical superstructures.

Moreover, it should also be noted that even a slight difference in positional isomeric *o-*H_2_MBA and *m-*H_2_MBA ligands can make structures and packing patterns different for Au_8_ nanoclusters (*R*-Au8d*versusR*-Au8e, Fig. S15†). In contrast to the case of *R*-Au8d, the weaker C–H⋯O interactions (average: 2.663 Å) are the dominant driving force for the formation of the helical superstructure of *R*-Au8e. This also implies a special role of the inserted (HCO_3_^−^)_2_ dimers in the hydrogen-bonded helixes of Au8c.

Furthermore, the chirality of crystalline-state Au8c was also investigated by CD spectroscopy. To eliminate the linear dichroism (LD) effect caused by crystal birefringent, the crystals of Au8c were adequately ground and mixed with KBr, and the mixtures were further pressed into tablets for testing. Compared to their CD spectra in diluted solution with three CD peaks, the CD spectra of crystalline-state Au8c are simpler in the same range. As shown in Fig. 5c, the *P*-helical *R*-Au8c displays two dominant Cotton effect bands at 368 nm (positive) and 517 nm (positive), while the *M*-helical *S*-Au8c affords a completely inverse CD spectrum. The discrepancy in the CD spectra of the solution and crystalline state can be related to the distinct intermolecular interactions. For Au8c, the hydrogen bonds and electrostatic interactions between individual Au_8_ nanoclusters and (HCO_3_^−^)_2_ dimers should play a key role in the crystalline state.

As indicated by ESI-MS, the most dominant species in CH_3_OH is monomeric 1a ([Au_8_(BINAP)_3_(*o-*HMBA)_2_]^2+^), accompanied by Au_8_ dimers with an extremely weak abundance, suggesting that the helical superstructures didn't exist in CH_3_OH (8.0 × 10^−6^ to 3.0 × 10^−4^ M), which is also evidenced by concentration-dependent IR and CD spectra of *R*-Au8c in CH_3_OH (Fig. S16 and S17†).

### Photoluminescence properties of Au8c

**Fig. 6:**
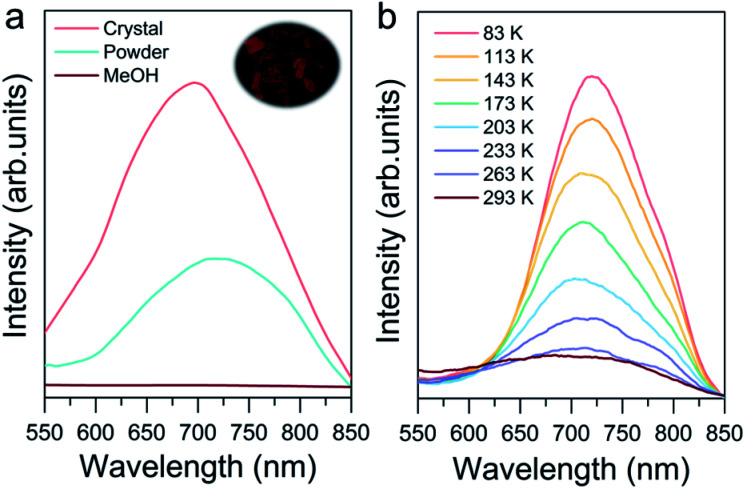
(a) Emission spectra of crystals (pink line), powder (cyan line) and CH_3_OH solution (brown line) of Au8c. Inset: photograph of Au8c crystals under green light excitation. (b) Temperature-dependent emission spectra of Au8c in the crystalline state (*λ*_ex_ = 365 nm).

Photoluminescence properties were also investigated to explore their potential applications such as in imaging, sensing and cell labeling.^76–78^ As shown in Fig. 6a, the crystalline state Au8c exhibited emission at 698 nm at room temperature under excitation at 365 nm. In contrast, the CH_3_OH solution of Au8c is nonemissive because of the chaos of Au_8_ nanoclusters in solution, which prevents the formation of ordered intermolecular interactions, suppressing the radiation decay route. For comparison, a powder sample was prepared by rotary evaporation of CH_3_OH solution of Au8c, and its luminescence intensity was weaker than that in the crystalline state along with a red-shift of 20 nm. This can be rationalized by the limited and chaotic intermolecular interactions in powder where a looser packing of the nanoclusters is formed.^79^ All of these results refer to a CIEE phenomenon in Au8c.^48,79^ The compact intermolecular hydrogen bonds as well as the electrostatic interactions distributed in Au8c might restrict the intra-molecular rotations and vibrations, which is attributed to the boosted luminescence. In summary, the special arrangement of Au8c nanoclusters in the crystal lattice plays the key role in determining its photoluminescence behavior. Additionally, we also observed temperature-dependent emissive behavior of Au8c in the crystalline state (Fig. 6b). Upon decreasing the temperature from 293 to 83 K, the intensity of the emission peak gradually increased along with a red-shift from 698 to 718 nm. A similar phenomenon has also been observed in other coinage metal clusters.^80^ We anticipate that Au8c with the near-infrared (NIR) luminescence feature can serve as a promising material for practical applications.

## Conclusions

In summary, we successfully synthesized a pair of atom-precise gold nanocluster enantiomers, *R*-Au8c and *S*-Au8c, through an elaborate selection of the hydrophobic chiral BINAP and hydrophilic achiral *o*-H_2_MBA ligands as protective ligands. Based on the single crystal structures, CD spectra in solution and DFT analysis, the chirality of individual Au8c nanoclusters is mainly contributed by the inherently chiral metallic kernel and BINAP ligands. Driven by intermolecular hydrogen bonds between (HCO_3_^−^)_2_ dimers and individual Au_8_ nanoclusters, *R*-Au8c and *S*-Au8c demonstrate *P*- and *M*-single stranded helical superstructures in the crystalline state, respectively. This work demonstrates a structural model for in-depth understanding of the self-assembly mechanisms of cluster-based helical superstructures and provides an effectively designed approach to construct atom-precise helical supramolecular materials. Future work will focus on exploring the potential applications of these helical superstructures and constructing more diverse helical superstructures.

## Data availability

All experimental and computational data associated with this article have been included in the main text and ESI.†

## Author contributions

D. S. conceived the manuscript; W. D. S. and K. S. performed the experiments; W. D. S., K. S., C. Z., Z. W., S. S. Z., J. M. D., L. F., Z. Y. G. and D. S. analyzed data, prepared figures and provided conceptual contributions; W. D. S., K. S., C. H. T. and D. S. wrote the manuscript with contributions from all co-authors.

## Conflicts of interest

The authors declare no competing financial interest.

## Supplementary Material

SC-013-D2SC03463H-s001

SC-013-D2SC03463H-s002
