# Supplementary material for: Bicarbonate insertion triggered self-assembly of chiral octa-gold nanoclusters into helical superstructures in the crystalline state
Source: Chem Sci. 2022 Aug 15;13(35):10523–31. doi: 10.1039/d2sc03463h (PMC9473528; doi:10.1039/d2sc03463h)
Supplement: SC-013-D2SC03463H-s001 [file SC-013-D2SC03463H-s001.pdf]

Electronic Supplementary Material (ESI) for Chemical Science.
This journal is © The Royal Society of Chemistry 2022

Supporting Information (SI)

Bicarbonate Insertion Triggered Self-Assembly of Chiral Octa-Gold Nanoclusters into Helical Superstructures in the Crystalline State

Wei-Dan Si,[a, ǂ] Kai Sheng,[b, ǂ] Chengkai Zhang,[a] Zhi Wang,[a] Shan-Shan Zhang,[a] Jian-Min Dou,[c] Lei Feng,[a] Zhi-Yong Gao,[d] Chen-Ho Tung,[a] and Di Sun[a,*]

[a]School of Chemistry and Chemical Engineering, State Key Laboratory of Crystal Materials, Shandong University, Ji'nan, 250100, People's Republic of China.
[b]School of Aeronautics, Shandong Jiaotong University, Ji'nan 250037, People's Republic of China.
[c]Shandong Provincial Key Laboratory of Chemical Energy Storage and Novel Cell Technology, and School of Chemistry and Chemical Engineering, Liaocheng University, Liaocheng 252000, People's Republic of China.
[d]School of Chemistry and Chemical Engineering, Henan Normal University, Henan Xinxiang 453007, People's Republic of China.
[ǂ]These authors contributed equally to this work.

Section 1. Experimental Section

I. Materials and regents

$[Au(SMe_2)Cl]$ was synthesized according to the literature method.[1] *R*/*S*-BINAP (Adamas-beta®) were purchased from Shanghai Titan Scientific Co., Ltd. Other chemicals and reagents employed in the syntheses were of analytical grade and employed as purchased without further purification. The solvents employed in all experiments was pure.

II. Synthesis

Synthesis of *R*-Au8c

The chiral ligand *R*-BINAP (12 mg, 0.02 mmol), *o*-H_2MBA (2 mg, 0.013 mmol) and $[Au(SMe_2)Cl]$ (6 mg, 0.02 mmol) were dissolved in a mixture of 6 mL CH_2Cl_2 and 1 mL CH_3OH. After stirring for 10 minutes, a freshly prepared $NaBH_4$ (3 mg in 1 mL water) was added dropwise under vigorous stirring at room temperature with the color of solution changing from pale yellow to orange and finally to brownish red. Then 30 μL Et_3N was added in the mixture and the reaction continued for 12 h in the dark. The solution was rotary vaporized to give a brownish red solid. The orange crystals of ***R*-Au8c** were obtained by diffusion CH_2Cl_2/*n*-hexane about three weeks at a yield of ~15% (based on Au). Combustion elementary analysis calculated (experimental) for it: ($C_{146}H_{106}Au_8O_4P_6S_2$): C, 46.76 (43.24%); H, 2.85 (2.61%). Selected IR peaks (cm^{-1}): 3731 (w), 3047 (w), 2912 (w), 2362 (s), 2341 (s), 1587 (m), 1574 (m), 1550 (w), 1435 (w), 1359 (m), 1091 (m), 872 (s), 813 (m), 741 (s), 687 (s), 574 (m), 522(s).

Synthesis of *S*-Au8c

The synthetic procedure of ***S*-Au8c** followed a similar to that of ***R*-Au8c,** except that the *S*-BINAP was employed instead of *R*-BINAP.

Synthesis of *R*-Au8d

The synthetic procedure of ***R*-Au8d** followed a similar to that of ***R*-Au8c,** except that the reaction were carried out in N_2 atmosphere.

Synthesis of *R*-Au8e

The synthetic procedure of ***R*-Au8e** followed a similar to that of ***R*-Au8c,** except that the *m*-H_2MBA (*m*-mercaptobenzoic acid) was employed instead of *o*-H_2MBA.

III. Physical measurements

Mass spectra (MS) were recorded on a Bruker impact II high definition mass spectrometer equipped with the cold spray ionization source (CryoSpray, Japan Thermal Engineering Co., Ltd.), quadrupole and time-of-flight (Q/TOF) modules in the positive ion mode. Typical measurement conditions are as follows: end plate offset = 500 V; dry gas = 4 L/min, nebulizer = 0.3 bar, capillary voltage = 4000 V, spray temperature = -40°C; sample flow rate = 500 μL/h; collision gas, N_2. Collision-induced dissociation (CID) mass spectrometry studies were performed by selecting the parent ion using a quadrupole mass filter and then colliding the ions with N_2 in the trap chamber. Samples were analyzed at different collision voltage. The reported *m/z* values represent monoisotopic mass of the most abundant peak within the isotope pattern. The data analyses of mass spectra were performed based on the isotope distribution patterns using Compass Data Analysis software (Version 4.4). UV-vis absorption spectra were recorded on a Thermo Scientific Evolution 220 UV-visible spectrophotometer. The circular dichroism (CD) spectra were recorded at room temperature with Applied Photophysics ChirascanV100 model. Luminescence spectra were carried out in an Edinburgh spectrofluorimeter (FLS920) at room temperature. Time-resolved luminescence lifetime measurements were performed on the same instrument by using a time-correlated single-photon counting technique. FTIR spectra were recorded on a Bruker Tensor II spectrophotometer (Bruker Optics GmbH, Ettlingen, Germany) utilizing a single attenuated total reflectance (ATR) accessory covering a wavenumber range from 400 to 4000 cm^{-1}. The final spectrum was the average of 32 scans accumulated using Bruker's Opus software 8.1, taken at 4 cm^{-1} resolution. The samples were measured under the same mechanical force pushing the samples in contact with the diamond window. Powder X-ray diffraction (PXRD) analyses were carried out on a microcrystalline powder using a Rigaku Oxford Diffraction XtaLAB Synergy-S diffractometer using Cu radiation (λ = 1.54184 Å).

The PXRD patterns were processed with the *CrysAlis^{Pro}* software suite[2] using the Powder function. Morphology of the sample and elemental composition analyses were measured using an SU-8010 field emission scanning electron microscope (FESEM; Hitachi Ltd., Tokyo, Japan) equipped with an Oxford-Horiba Inca XMax50 energy dispersive X-ray spectroscopy (EDS) attachment (Oxford Instruments Analytical, High Wycombe, England).

X-ray Crystallography

Single crystals of ***R*-Au8c** and ***S*-Au8c** with appropriate dimensions were chosen under an optical microscope and quickly coated with high vacuum grease (Dow Corning Corporation) to prevent decomposition. The crystals were mounted on a CryoLoop™ and cell parameters and intensity data were recorded on a Rigaku Oxford Diffraction XtaLAB Synergy-S diffractometer equipped with a HyPix-6000HE Hybrid Photon Counting (HPC) detector operating in shutterless mode and an Oxford Cryosystems Cryostream 800 Plus using Cu Kα (λ = 1.54184 Å) for ***R*-Au8c** and ***S*-Au8c** at 100 K from PhotonJet micro-focus X-ray Source. Data were processed using the *CrystAlis*^{Pro} software suite.[2] The intensity data and cell parameters of ***R*-Au8d** and ***S*-Au8e** were recorded at 100 K on Bruker D8 VENTURE diffractometer with an Incoatec IμS 3.0 Cu EF microfocus source (55W, Cu Kα, λ = 1.54178 Å) equipped with a PHOTON III C28 detector and an Oxford Cryosystems CryostreamPlus 800 open-fow N_2 cooling device. The raw frame data were processed using SAINT and SADABS to yield the reflection data file.[3]

These structures were solved using the charge-flipping algorithm, as implemented in the program *SUPERFLIP*[4] and refined by full-matrix least-squares techniques against $F_o{}^2$ using the SHELXL program[5] through the OLEX2 interface.[6] Hydrogen atoms at carbon were placed in calculated positions and refined isotropically by using a riding model. Appropriate restraints or constraints were applied to the geometry and the atomic displacement parameters of the atoms in the cluster. All structures were examined using the Addsym subroutine of PLATON[7] to ensure that no additional symmetry could be applied to the models. Pertinent crystallographic data collection and refinement parameters are collated in Table S1 and Table S3. Selected bond lengths are collated in Table S2.

IV. Computational Studies

DFT calculations were performed with the Gaussian 16 suite of programs.[8] For the optimizations of the cationic nanoclusters ***R*-Au8c** and ***S*-Au8c**, the gradient-corrected MPW1PW91 exchange correlation functional, based on the generalized gradient approximation (GGA) was utilized;[9] LanL2DZ (Los Alamos effective core potential double-ζ) basis set was employed for Au atoms, augmented with f-type polarization functions and 6-31G(d) basis set was used for C, H, O, S and P atoms. The MPW1PW91 functional is expected to be more suitable for the second and third row transition metal systems.[10] The LANL2DZ basis set containing relativistic effects has been shown to predict accurately the structure of Au nanoclusters.[11, 12] Spin-restricted calculations were used for geometry optimization. Harmonic frequencies were then calculated to characterize the stationary points as equilibrium structures with all real frequencies, and to evaluate zero-point energy (ZPE) corrections. The TD-DFT calculations were performed to get the most probable transitions and the orbitals corresponding to the main peaks in the calculated electronic spectrum. A total of 350 singlet states were chosen in the calculations. The root is set as 1 in the TD-DFT calculations. Data for orbital composition analysis with Mulliken partition are from Gaussian 16 calculations and further processed with Multiwfn software.[13] The most probable transitions were determined based on the oscillator strength values and weights. The optical absorption and CD spectra were convoluted with a Gaussian line shape with a half-width at half-height of 0.20 eV.

Section 2. Supplementary Figures

Fig. S1. Photograph for crystals of Au8c in mother liquor (left) and amplified photograph (right).

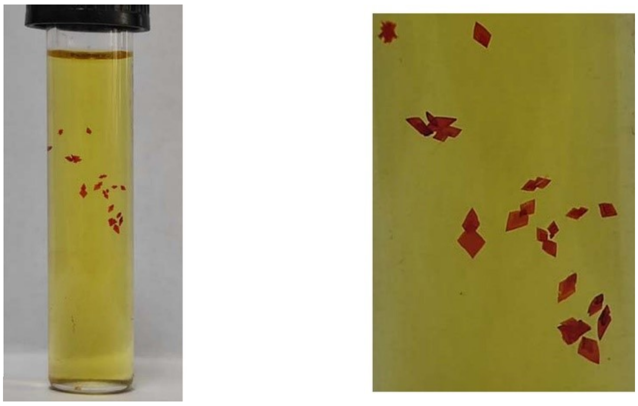

Fig. S2. Single-crystal structure of *R*-Au8d nanocluster. Color labels: pink, Au; yellow, S; orange, P; gray, C; red, O; white, H.

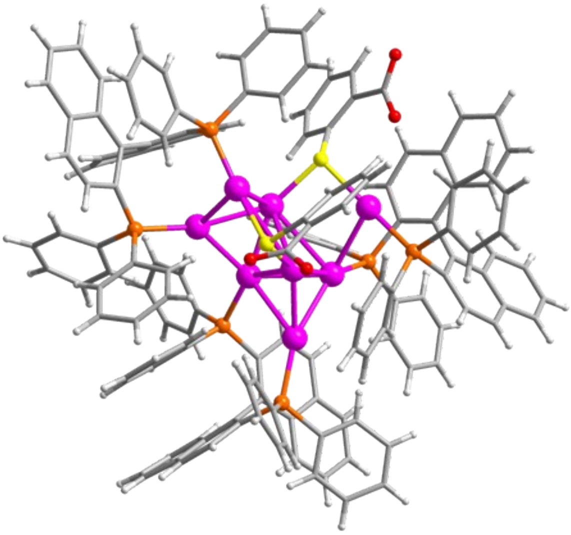

R-Au8d

Fig. S3. Single-crystal structure of *R*-Au8e nanocluster. Color labels: pink, Au; yellow, S; orange, P; gray, C; red, O; white, H.

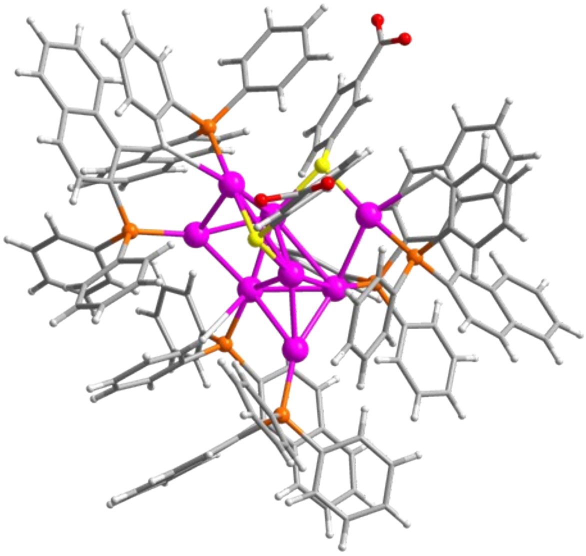

R-Au8e

Fig. S4. IR spectra of Au8c (pink line) and $NaHCO_3$ (cyan line).

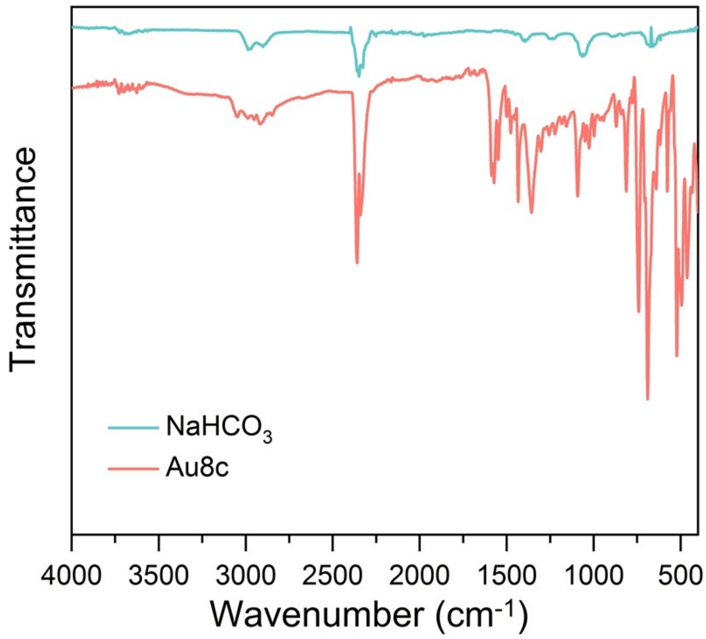

Fig. S5. The PXRD patterns of *R*-Au8c (a) and *S*-Au8c (b).

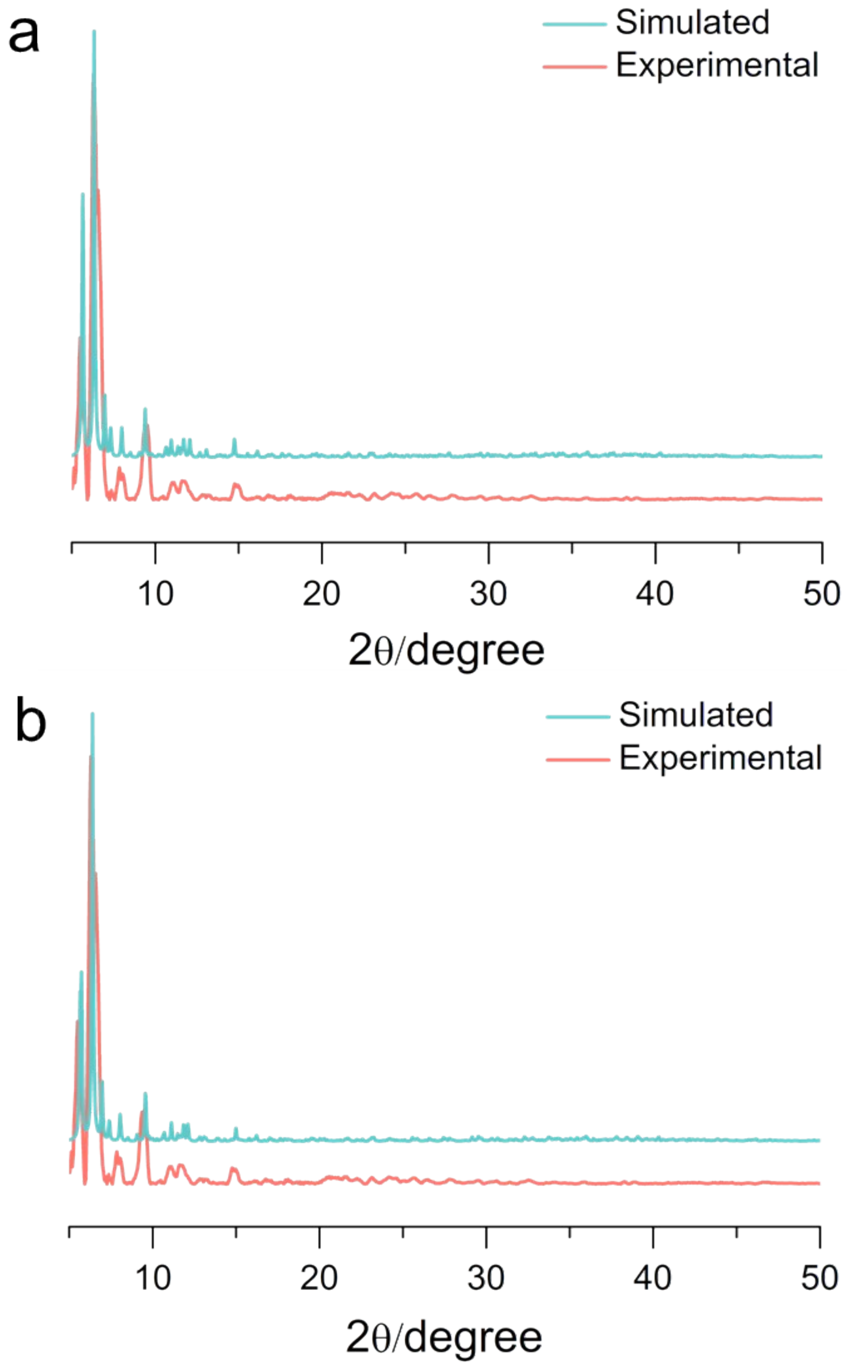

Fig. S6 The Au_8 kernels with coordinated atoms in *R*-Au8c (left) and *S*-Au8c (right). Color labels: pink, green, Au; yellow, S; orange, P.

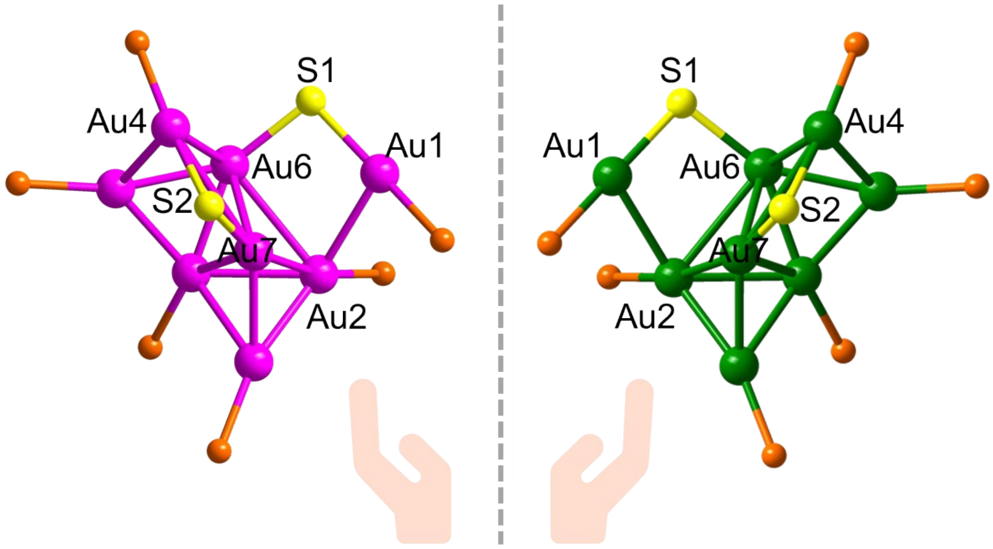

Fig. S7. Optimized structures of *R*-Au8c (a) and *S*-Au8c (b), and selected weak Au···H–C interactions.

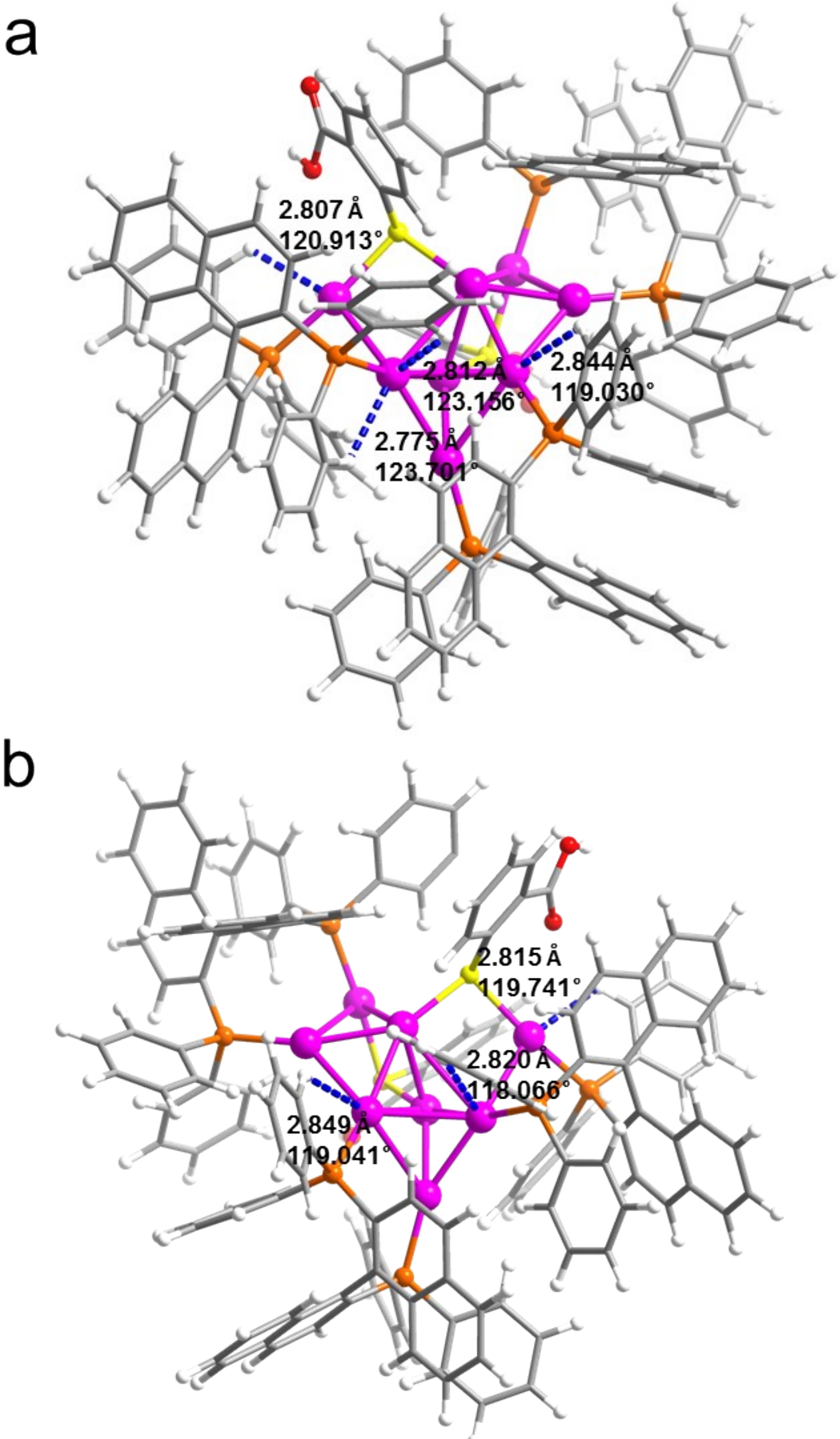

Fig. S8. Noncovalent interaction (NCI) analysis for *R*-Au8c nanoclusters. The green and/or cyan clouds can be assigned as the space where Au···H interactions happened, and cyan clouds revealed stronger interactions. (green, Au; dark green, P; yellow, S; cyan, C; red, O; white, H.)

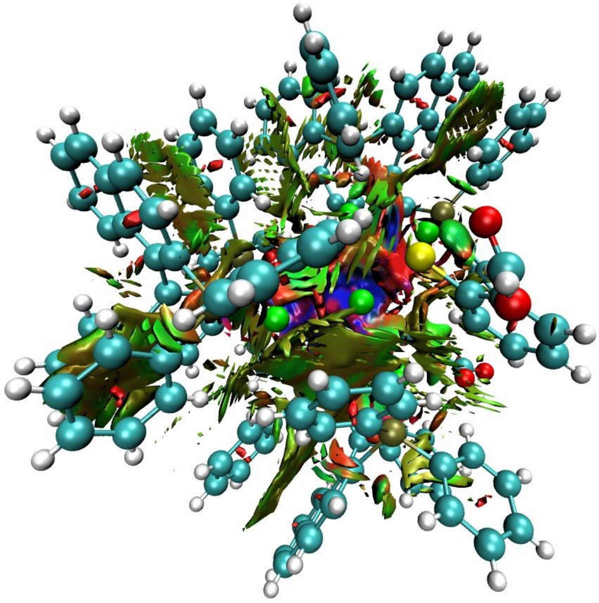

Fig. S9. Comparison of the experimental (pink line) and simulated (cyan line) isotopic patterns of 1c, 1l-1q.

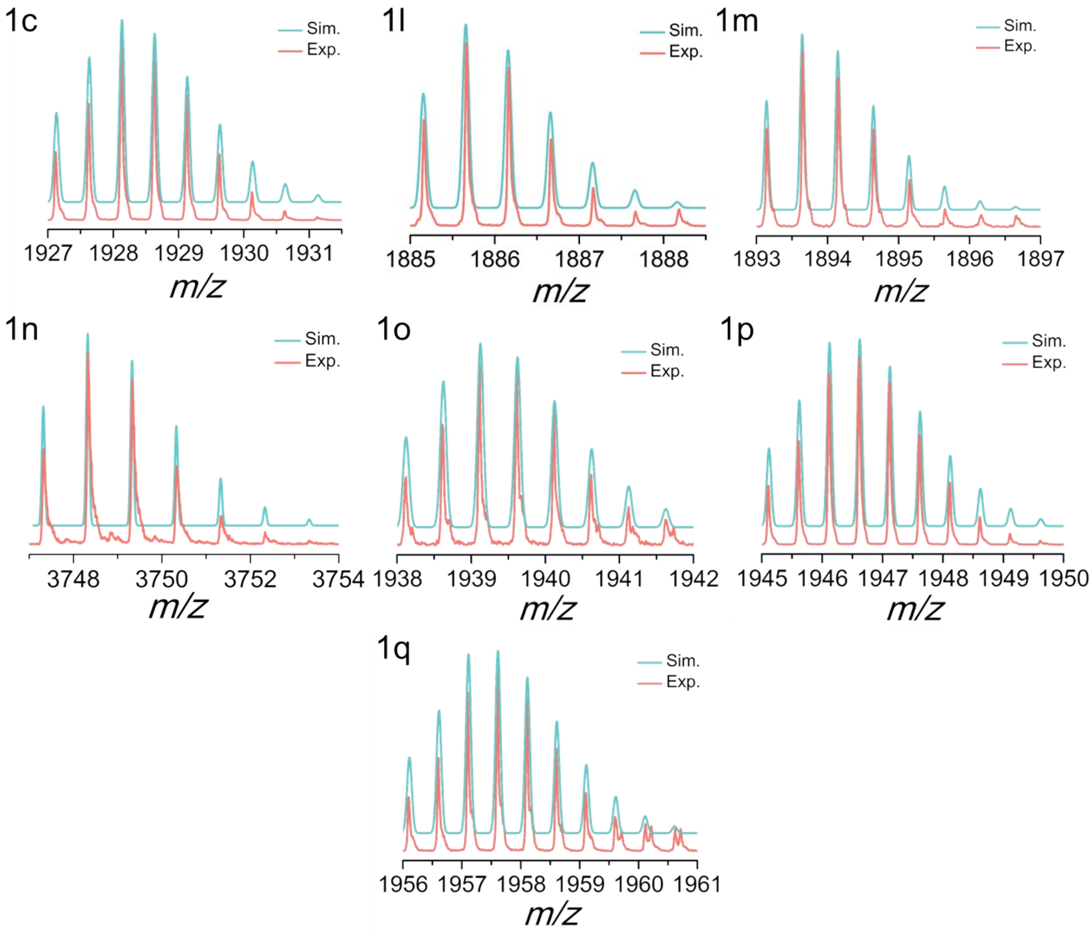

Fig. S10. Relative intensity of the Au8c resulting fragment ions collected at the voltage range of 0-55eV (1a, 1d-1k) (a). The proposed gas-phase dissociation pathways for [$Au_8(BINAP)_3$(*o*-HMBA)$_2$]$^{2+}$ (b).

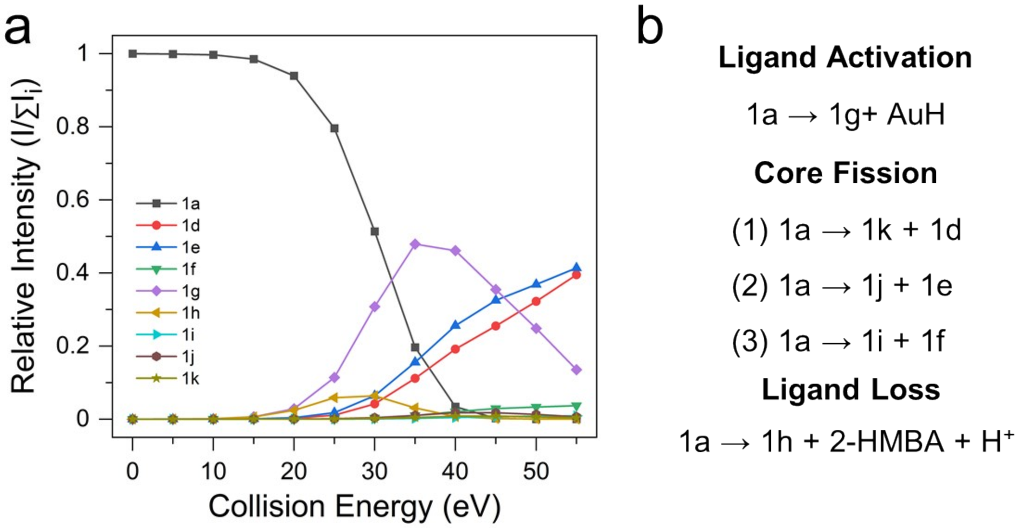

Fig. S11. Comparison of the experimental (pink line) and simulated (cyan line) isotopic patterns of 1d-1k.

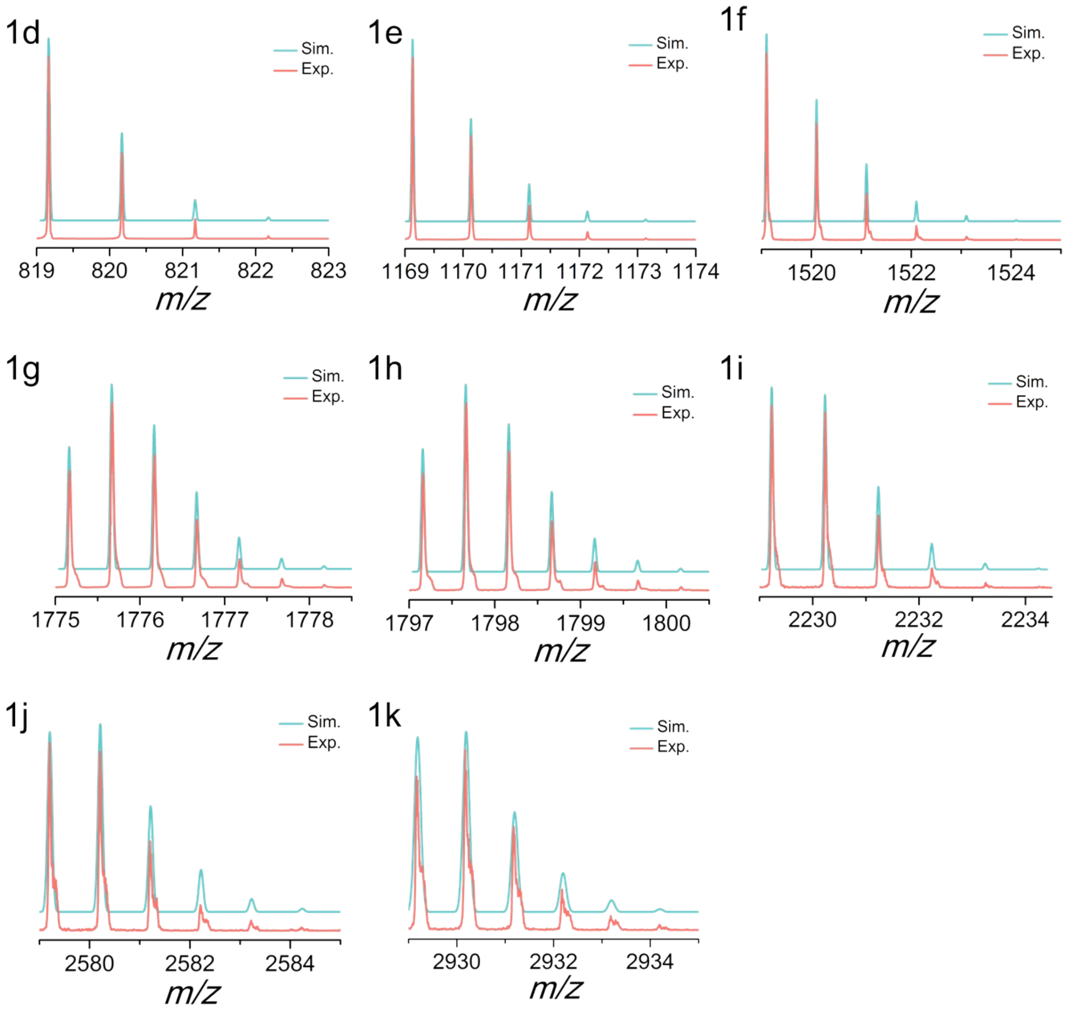

Fig. S12. Time-dependent UV-vis absorption spectra of Au8c in CH_3OH.

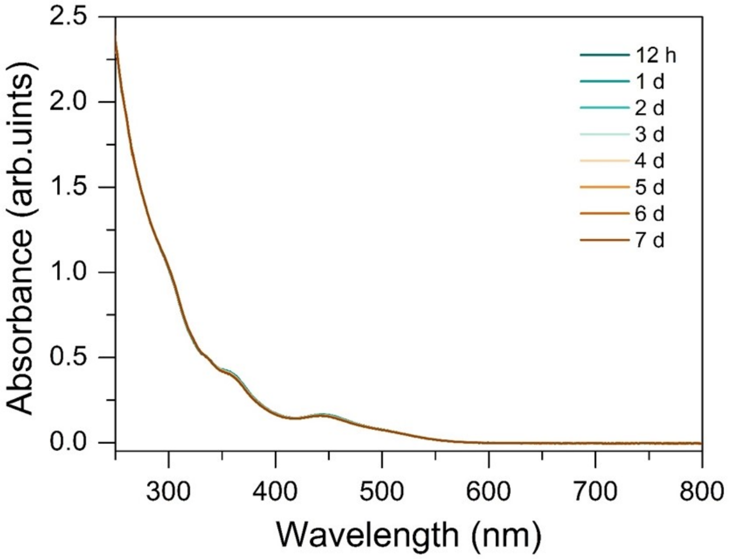

Fig. S13. Selected calculated orbitals of *R*-Au8c.

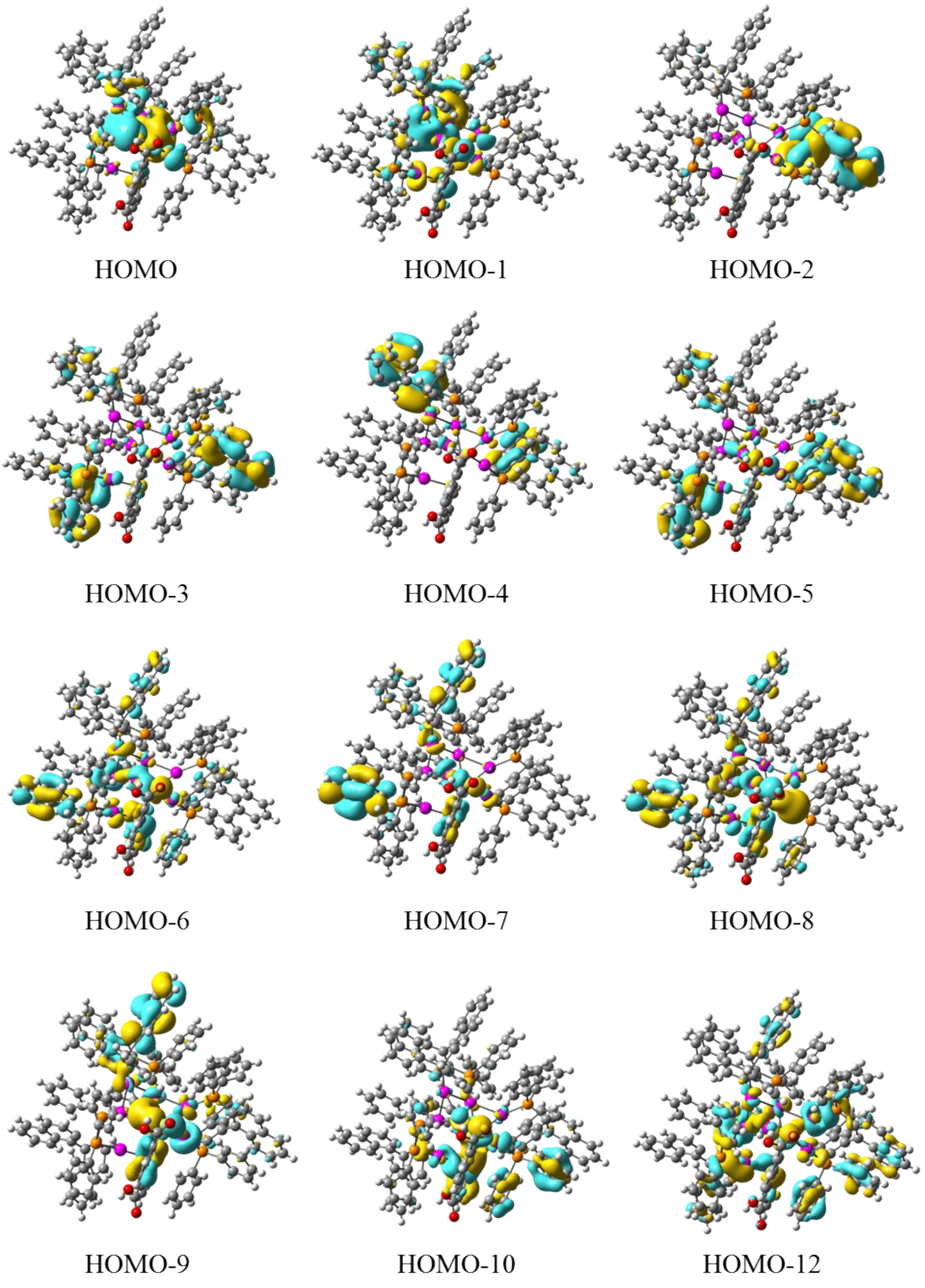

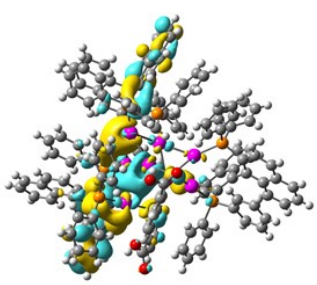

LUMO

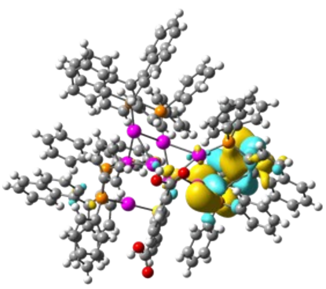

LUMO+1

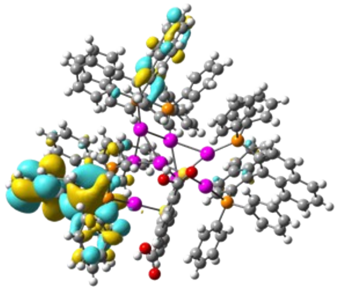

LUMO+2

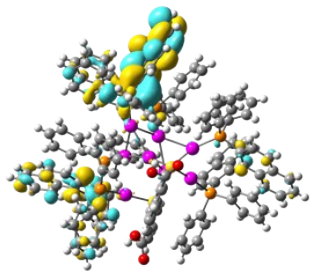

LUMO+3

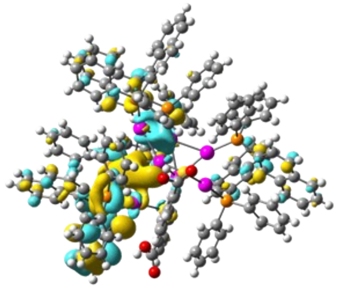

LUMO+4

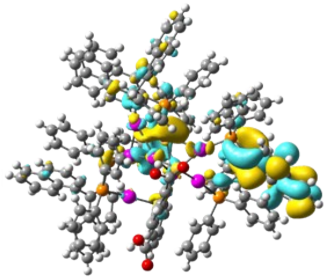

LUMO+5

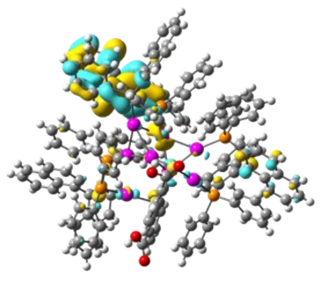

LUMO+6

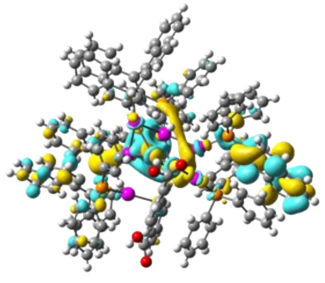

LUMO+7

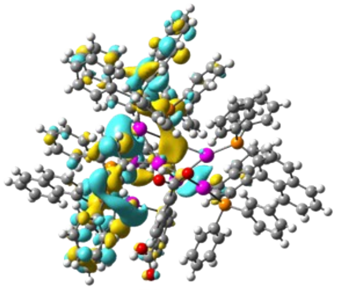

LUMO+8

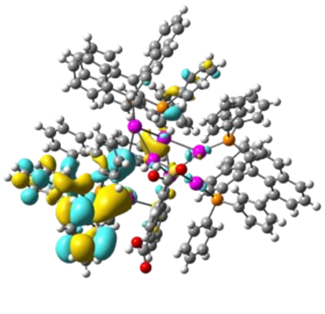

LUMO+9

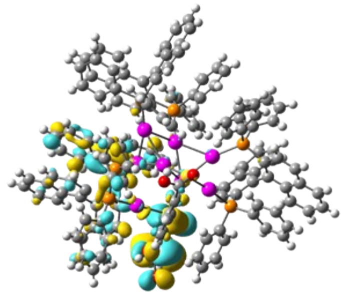

LUMO+10

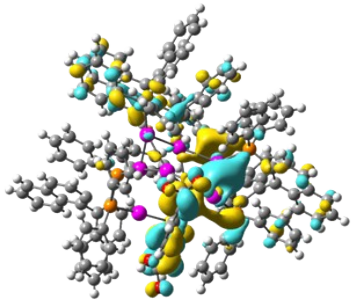

LUMO+11

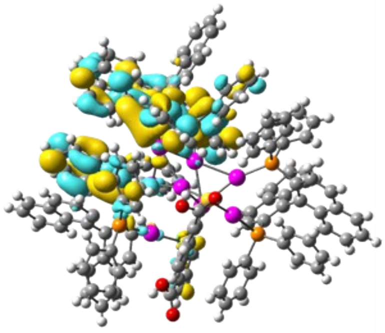

LUMO+13

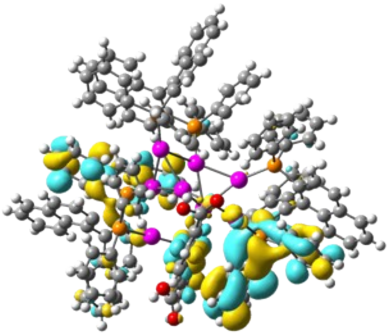

LUMO+23

Fig. S14. The C–H···O and C–H···π interactions in *R*-Au8d lead to the helical packing along *b* axis.

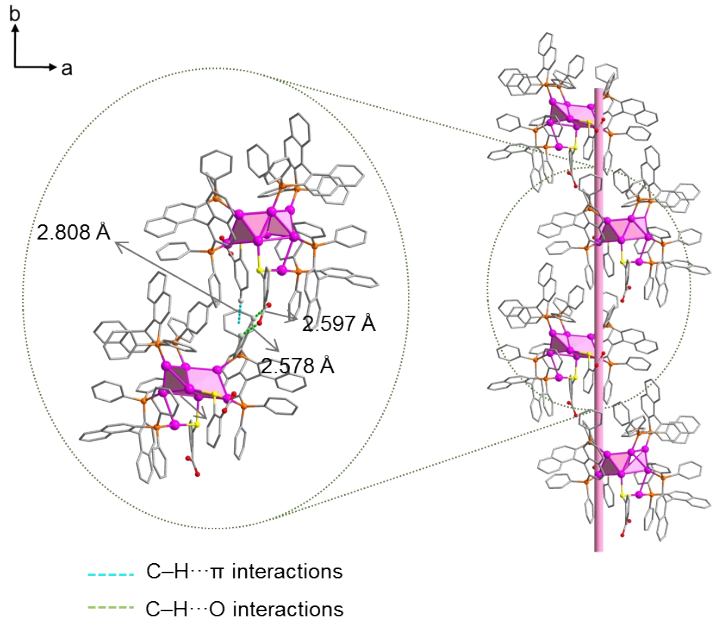

Fig. S15. The C–H···O interactions in *R*-Au8e lead to the helical packing along *b* axis.

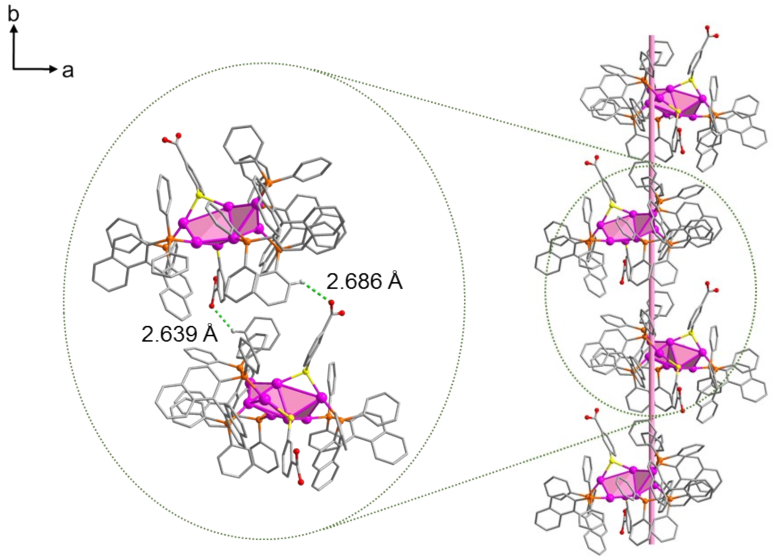

Fig. S16. CD spectra of *R*-Au8c in different concentrations of CH_3OH (8×10^{-6} M to 3×10^{-4} M).

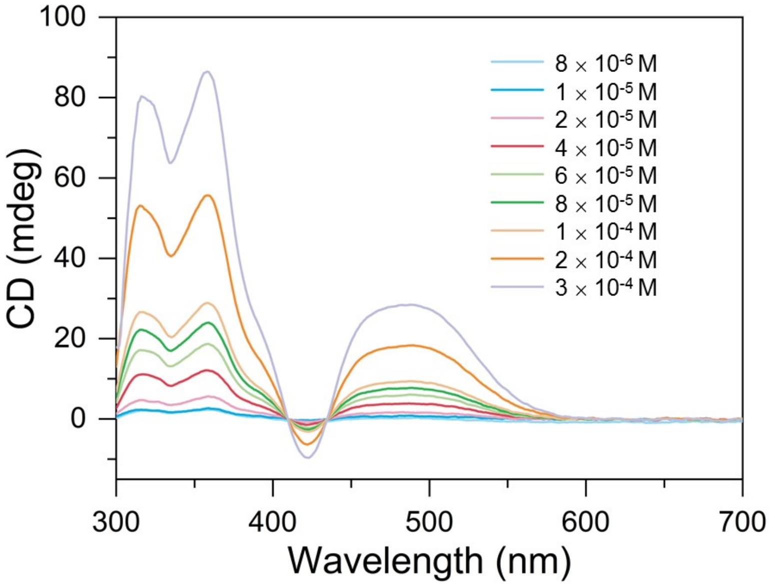

Upon increasing the ***R*-Au8c** concentration, the characteristic CD peaks did not shift, suggesting that individual ***R*-Au8c** nanoclusters may have not been assembled in the range of 8.0×10^{-6} to 3.0×10^{-4} M.

Fig. S17. IR spectra of *R*-Au8c in different concentrations of CH_3OH (8 × 10^{-6} M to 3× 10^{-4} M).

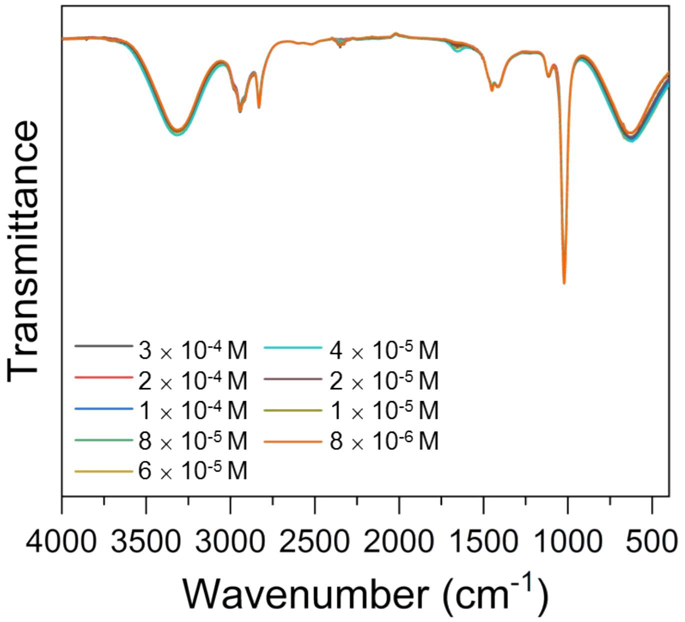

Upon increasing the ***R*-Au8c** concentration, the IR peaks did not shift, suggesting that individual ***R*-Au8c** nanoclusters may have not been assembled in the range of 8.0 × 10^{-6} to 3.0 × 10^{-4} M.

Fig. S18. The bright-field image (left) of Au8c crystals and its fluorescent image (right) under green light excitation (Murzider MSD520T).

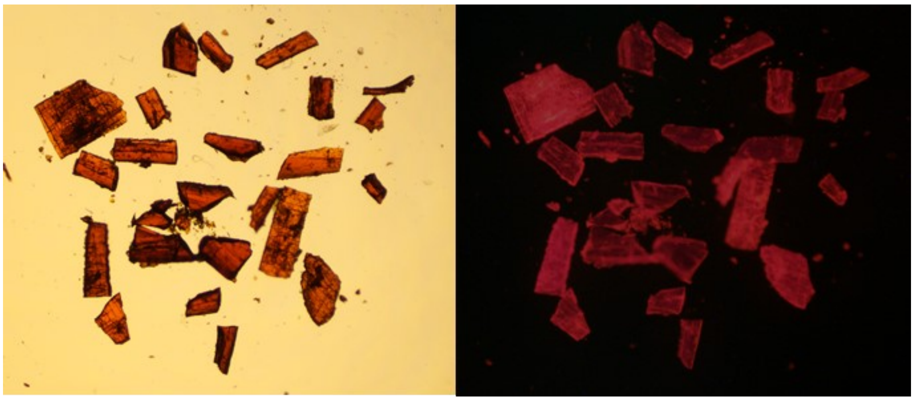

Fig. S19. The luminescent lifetime of Au8c in crystalline state at 293 K (a) and 83 K (b).

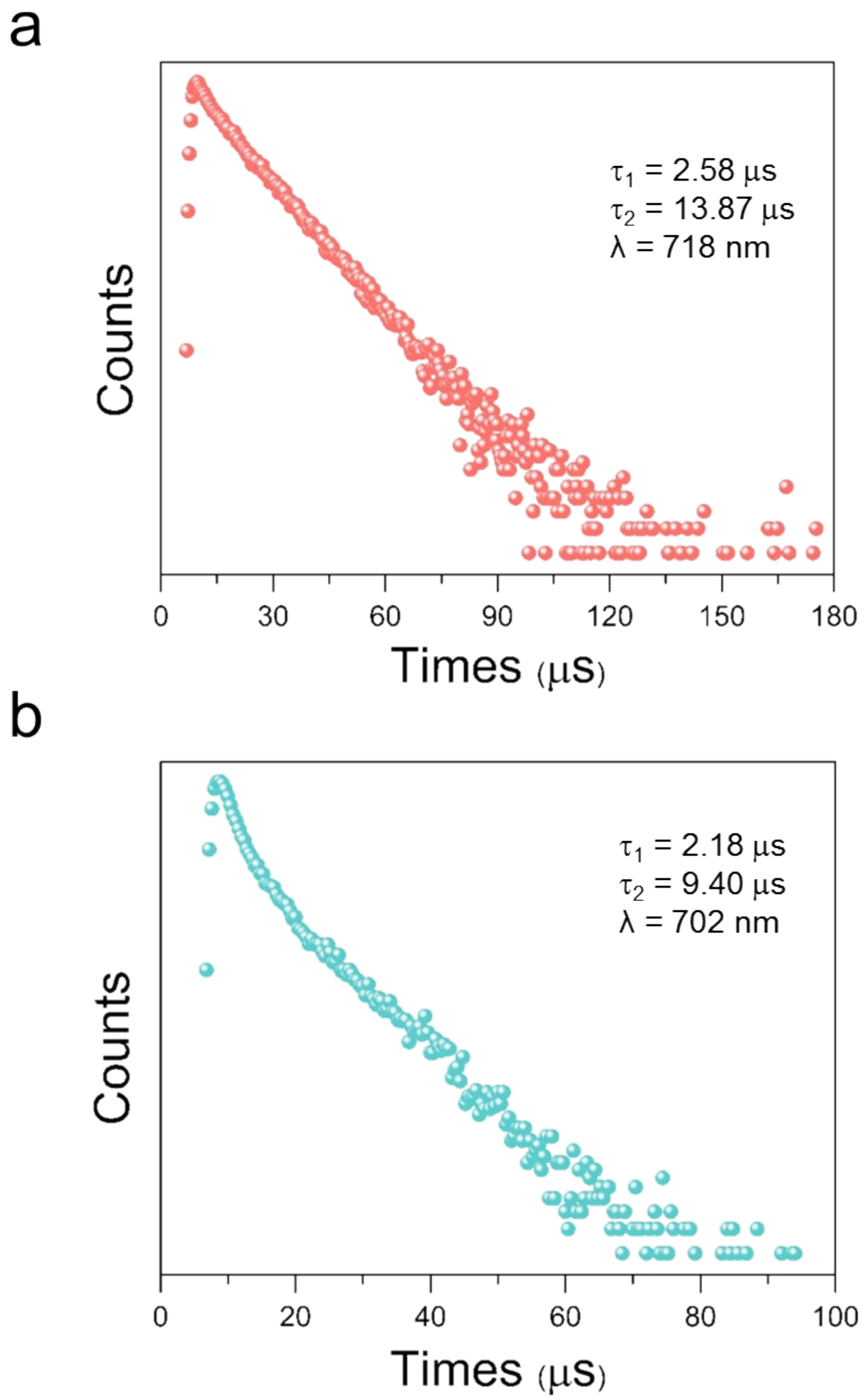

Fig. S20. The crystalline state UV-vis spectrum of Au8c was measured with the diffuse reflectance mode.

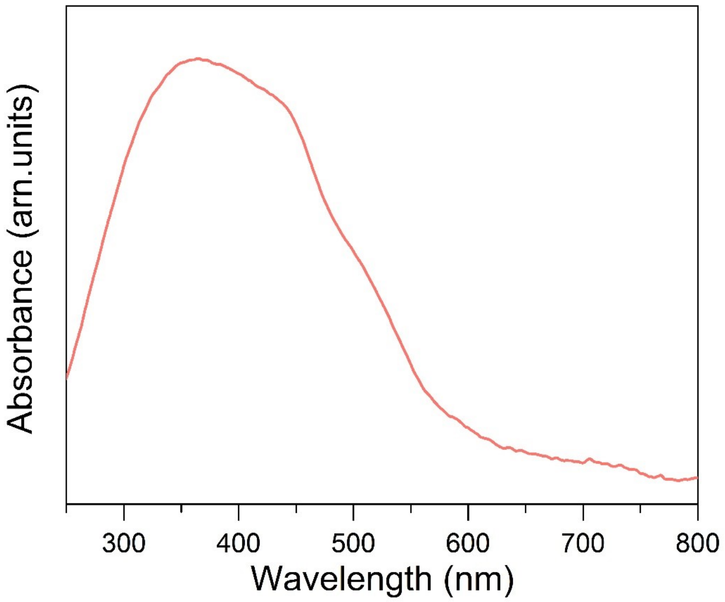

Fig. S21. Perspective of stacking in *R*-Au8c along the crystallographic *a*, *b* and *c* axis.

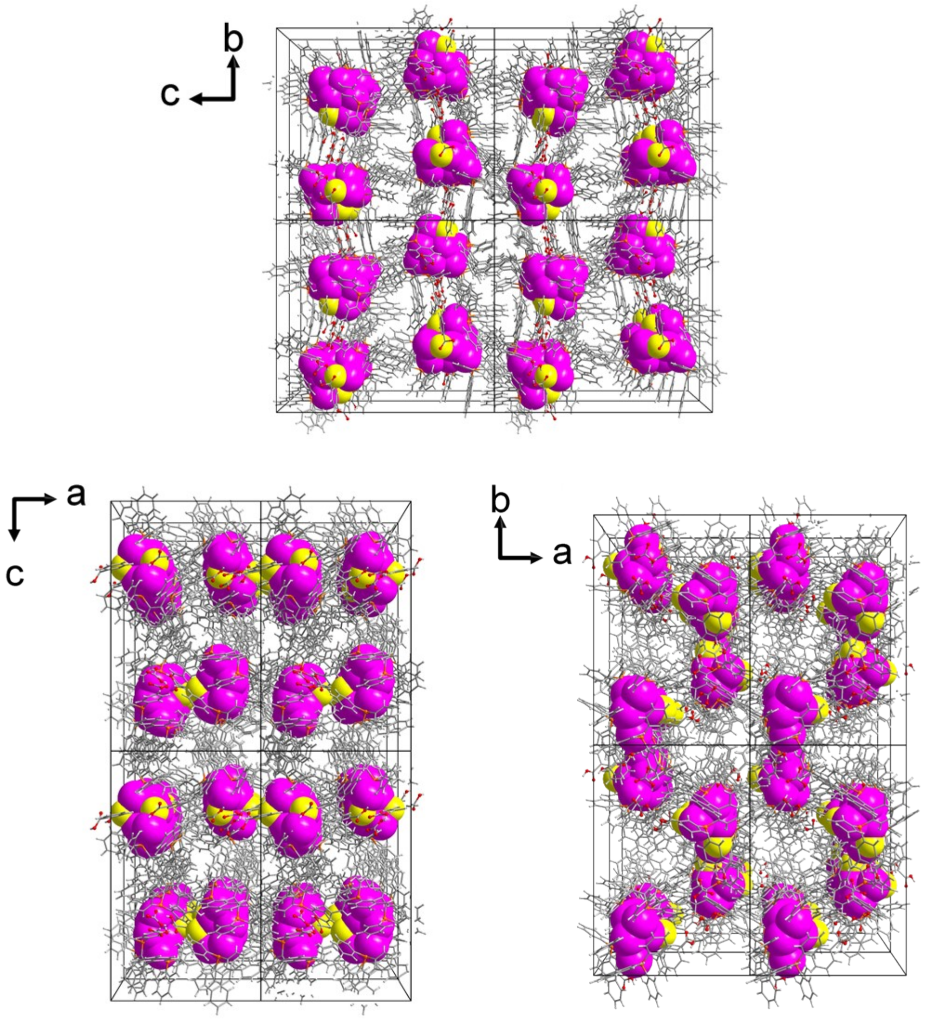

Fig. S22. Perspective of stacking in *S*-Au8c along the crystallographic *a*, *b* and *c* axis.

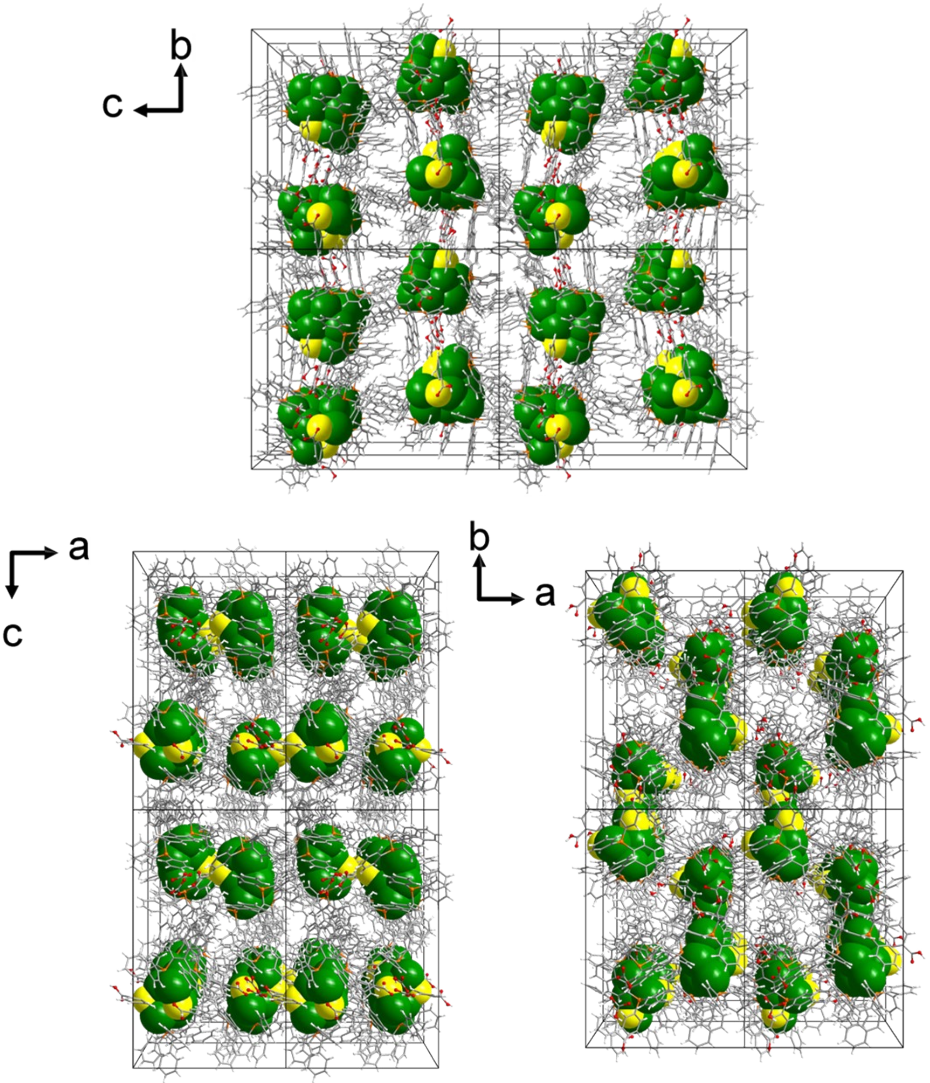

Fig. S23. SEM and elemental mapping images of Au8c.

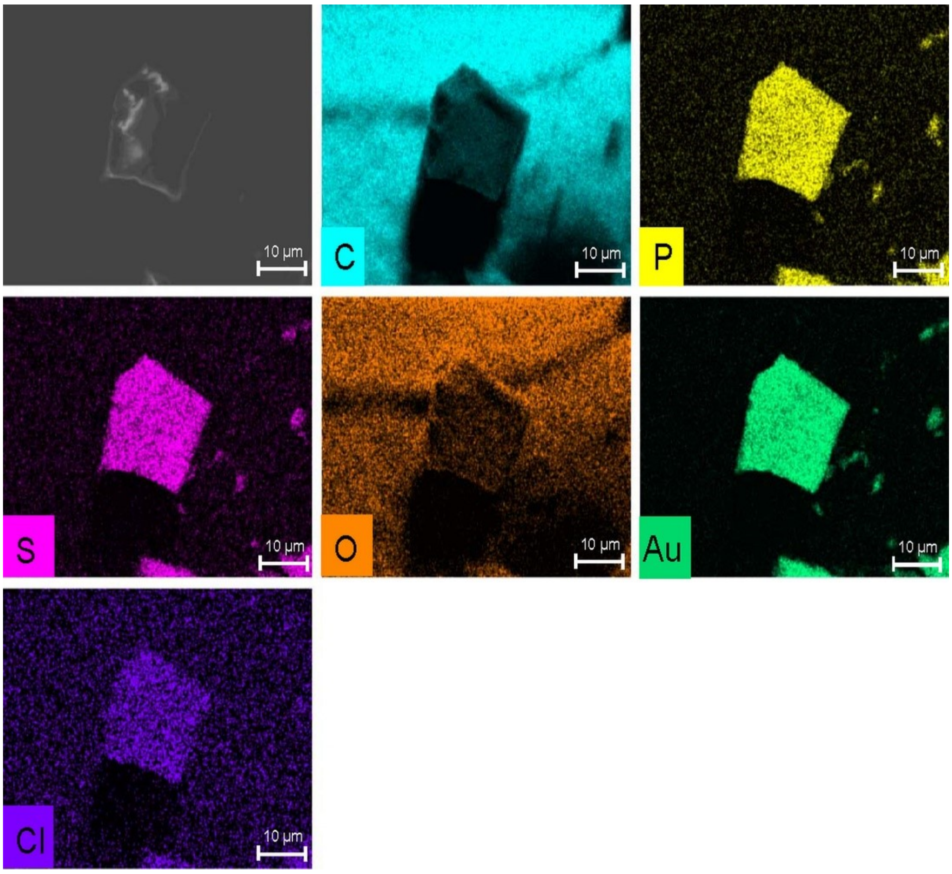

Fig. S24. The surface of *R*-Au8c calculated via 3V Volume Assessor program[14] by rolling a virtual probe (1.0 Å) on the surface viewed along six different orientations. The calculated surface area is 1689 Å^2.

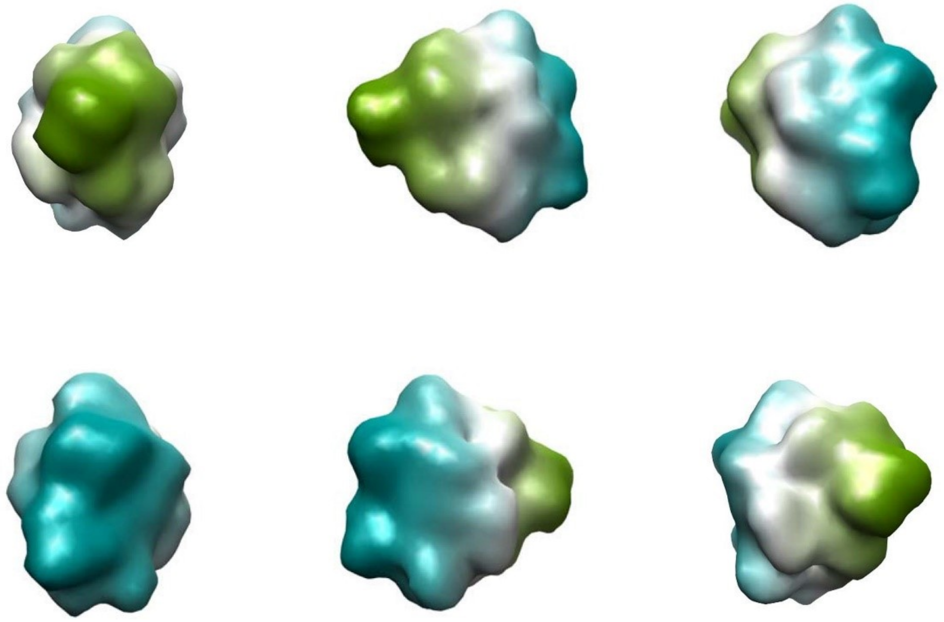

Fig. S25. The surface of *S*-Au8c calculated via 3V Volume Assessor program[14] by rolling a virtual probe (1.0 Å) on the surface viewed along six different orientations. The calculated surface area is 1698 Å^2.

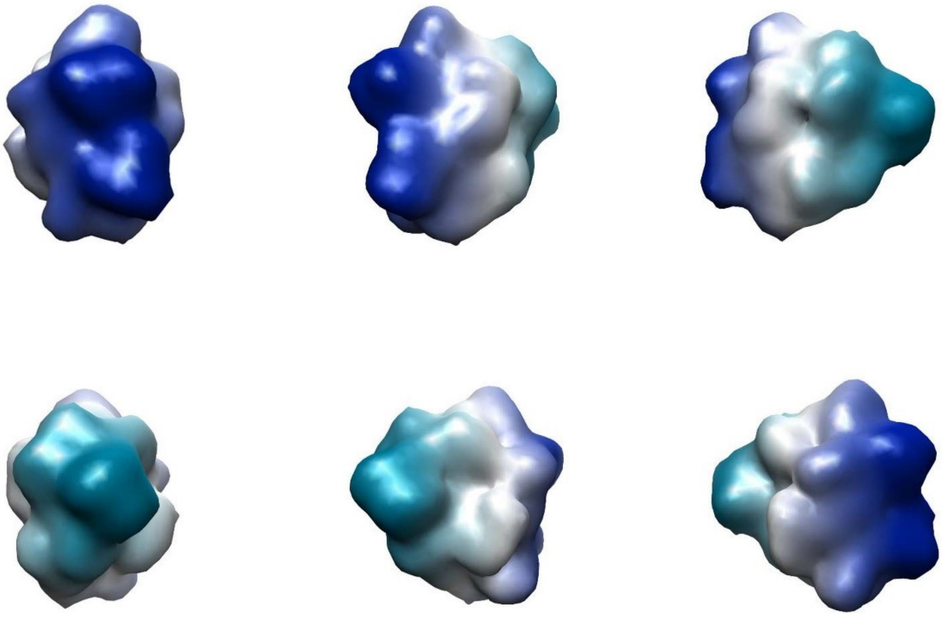

Section 3. Supplementary Tables

Table S1. Crystal data and structure refinement for *R*-Au8c and *S*-Au8c.

Identification code	***R*-Au8c**	***S*-Au8c**
Empirical formula	$C_{148}H_{108}Au_8O_{10}P_6S_2$	$C_{148}H_{108}Au_8O_{10}P_6S_2$
Formula weight	3782.01	3872.01
Temperature/K	100.01(10)	99.99(10)
Crystal system	orthorhombic	orthorhombic
Space group	$P2_12_12_1$	$P2_12_12_1$
a/Å	18.8119(2)	18.4744(2)
b/Å	27.6970(2)	27.7085(3)
c/Å	31.3398(3)	31.2344(3)
α/°	90	90
β/°	90	90
γ/°	90	90
Volume/Å^3	16329.1(3)	15988.8(3)
Z	4	4
ρ_{calc}g/cm^3	1.575	1.609
μ/mm^{-1}	14.326	14.631
F(000)	7320.0	7320.0
2Θ range for data collection/°	5.48 to 134.154	5.658 to 134.158
Index ranges	$-22 \leq h \leq 15$, $-32 \leq k \leq 33$, $-37 \leq l \leq 34$	$-8 \leq h \leq 22$, $-33 \leq k \leq 26$, $-28 \leq l \leq 37$
Radiation, CuKα	1.54184	1.54184
Reflections collected	70011	48414
Independent reflections	28706 [R_{int} = 0.0863, R_{sigma} = 0.0788]	26263 [R_{int} = 0.0641, R_{sigma} = 0.0742]
Data/restraints/parameters	28706/1077/1504	26263/1057/1486
Goodness-of-fit on F^2	1.076	1.054
Final R indexes [I>=2σ (I)]	R_1 = 0.0815, wR_2 = 0.2257	R_1 = 0.0672, wR_2 = 0.1687
Final R indexes [all data]	R_1 = 0.0918, wR_2 = 0.2437	R_1 = 0.0882, wR_2 = 0.1882
Largest diff. peak/hole / e Å^{-3}	3.33/-3.18	2.37/-1.68
Flack parameter	0.012(12)	0.041(8)

Table S2. Selected bond lengths (Å) and angle (°) for *R*-Au8c and S-Au8c.

***R*-Au8c**			
Au1-Au2	2.8983(15)	Au4-S2	2.397(10)
Au1-S1	2.337(7)	Au5-Au6	2.6480(13)
Au1-P1	2.284(8)	Au5-Au8	2.6813(15)
Au2-Au3	2.6912(15)	Au5-P4	2.279(6)
Au2-Au4	2.655(2)	Au6-Au7	3.0030(17)
Au2-Au5	2.9164(14)	Au6-Au8	2.6620(14)
Au2-Au6	3.2112(15)	Au6-S1	2.384(5)
Au2-P2	2.316(9)	Au7-Au8	2.9528(15)
Au3-Au4	2.7314(19)	Au7-S2	2.372(11)
Au3-Au5	2.8305(15)	Au7-P6	2.297(11)
Au3-P3	2.272(7)	Au8-P5	2.293(7)
Au4-Au5	2.8837(16)	P1-Au1-S1	169.2(4)
Au4-Au6	2.8388(16)	P6-Au7-S2	169.9(3)
***S*-Au8c**			
Au1-Au2	2.9006(15)	Au4-S2	2.378(8)
Au1-S1	2.320(6)	Au5-Au6	2.6512(14)
Au1-P1	2.263(9)	Au5-Au8	2.6759(16)
Au2-Au3	2.6902(15)	Au5-P4	2.287(6)
Au2-Au4	2.6525(17)	Au6-Au7	2.9760(16)
Au2-Au5	2.9208(14)	Au6-Au8	2.6669(14)
Au2-Au6	3.2111(15)	Au6-S1	2.363(5)
Au2-P2	2.332(8)	Au7-Au8	2.9721(15)
Au3-Au4	2.7277(18)	Au7-S2	2.367(10)
Au3-Au5	2.8263(16)	Au7-P6	2.278(9)
Au3-P3	2.278(8)	Au8-P5	2.302(7)
Au4-Au5	2.8924(14)	P1-Au1-S1	169.2(3)
Au4-Au6	2.8440(16)	P6-Au7-S2	169.8(3)

Table S3. Assignment of the key species of Au8c in CH_3OH.

species	assignment	Sim.	Exp.
**1a**	$[Au_8(BINAP)_3(o\text{-HMBA})_2]^{2+}$	1874.68	1874.67
**1b**	$[Au_{16}(BINAP)_6(o\text{-HMBA})_3(o\text{-MBA})_1]^{3+}$	2499.56	2499.55
**1c**	$[Au_8(BINAP)_3(o\text{-HMBA})_1(o\text{-MBA})_1]^{+}$	3748.34	3748.32
**1l**	$[Au_8(BINAP)_3(o\text{-HMBA})_1(o\text{-MBA})_1Na]^{2+}$	1885.67	1885.66
**1m**	$[Au_8(BINAP)_3(o\text{-HMBA})_1(o\text{-MBA})_1K]^{2+}$	1893.65	1893.64
**1n**	$[Au_8(BINAP)_3(o\text{-HMBA})_1(o\text{-MBA})_1Na(CH_2Cl_2)]^{2+}$	1928.13	1928.12
**1o**	$[Au_8(BINAP)_3(o\text{-MBA})_2Na_2\ (CH_2Cl_2)]^{2+}$	1939.12	1939.11
**1p**	$[Au_8(BINAP)_3(o\text{-HMBA})_2NaCl(CH_2Cl_2)]^{2+}$	1946.62	1946.61
**1q**	$[Au_8(BINAP)_3(o\text{-HMBA})_1(o\text{-MBA})_1Na_2Cl(CH_2Cl_2)]^{2+}$	1957.61	1957.60

Table S4. Assignment of the key species of Au8c in CH_3OH identified by CID-MS.

species	assignment	Exp.	Sim.
**1d**	$[Au_1(BINAP)_1]^+$	819.16	819.15
**1e**	$[Au_2(BINAP)_1(o\text{-}HMBA)_1]^+$	1169.13	1169.12
**1f**	$[Au_3(BINAP)_1(o\text{-}HMBA)_2]^+$	1519.10	1519.09
**1g**	$[Au_7(BINAP)_2(BINAP\text{-}H)_1(o\text{-}HMBA)_2]^{2+}$	1775.68	1775.67
**1h**	$[Au_8(BINAP)_3(o\text{-}MBA)_1]^{2+}$	1797.67	1797.66
**1i**	$[Au_5(BINAP)_2]^+$	2229.24	2229.23
**1j**	$[Au_6(BINAP)_2(o\text{-}HMBA)_1]^+$	2580.21	2580.20
**1k**	$[Au_7(BINAP)_2(o\text{-}HMBA)_2]^+$	2930.18	2930.17

Table S5. The excited states, energies (in eV and nm), oscillator strengths, weights and transitions with the most weights involved in cluster Au8c obtained by TD-DFT calculations.

Excited state	Energy (eV)	nm	Oscillator strength (a.u.)	Weight	Most weighted transitions	Nature of transition
1	2.5994	476.97	0.0326	0.6732	HOMO→LUMO	M$_{(Au)}$L$_{(BINAP)}$CT
				0.1436	HOMO→LUMO+4	M$_{(Au)}$L$_{(BINAP)}$CT
2	2.7755	446.71	0.0416	0.6084	HOMO→LUMO+1	M$_{(Au)}$L$_{(BINAP)}$CT
				0.2084	HOMO→LUMO+5	M$_{(Au)}$L$_{(BINAP)}$CT mixed M$_{(Au)}$M$_{(Au)}$CT
				0.1827	HOMO→LUMO+7	M$_{(Au)}$L$_{(BINAP)}$CT mixed M$_{(Au)}$M$_{(Au)}$CT
4	2.9373	422.10	0.0125	0.4192	HOMO→LUMO+3	M$_{(Au)}$L$_{(BINAP)}$CT mixed L$_{(\mathit{o}\text{-HMBA})}L_{(BINAP)}$CT
				0.3785	HOMO→LUMO+5	M$_{(Au)}$L$_{(BINAP)}$CT mixed M$_{(Au)}$M$_{(Au)}$CT
6	3.0272	417.79	0.0142	0.5960	HOMO→LUMO+2	M$_{(Au)}$L$_{(BINAP)}$CT
				0.2504	HOMO→LUMO+4	M$_{(Au)}$L$_{(BINAP)}$CT
11	3.2506	381.42	0.0215	0.4917	HOMO-1→LUMO+3	M$_{(Au)}$L$_{(BINAP)}$CT mixed L$_{(\mathit{o}\text{-HMBA})}L_{(BINAP)}$CT
				0.3364	HOMO-1→LUMO+4	M$_{(Au)}$L$_{(BINAP)}$CT mixed L$_{(\mathit{o}\text{-HMBA})}L_{(BINAP)}$CT
14	3.3595	369.05	0.0278	0.4949	HOMO-1→LUMO+2	M$_{(Au)}$L$_{(BINAP)}$CT mixed L$_{(\mathit{o}\text{-HMBA})}L_{(BINAP)}$CT
				0.3472	HOMO→LUMO+8	M$_{(Au)}$L$_{(BINAP)}$CT mixed M$_{(Au)}$M$_{(Au)}$CT
16	3.4272	361.77	0.0273	0.3348	HOMO→LUMO+9	M$_{(Au)}$L$_{(BINAP)}$CT
				0.3052	HOMO→LUMO+10	M$_{(Au)}$L$_{(BINAP)}$CT mixed M$_{(Au)}$L$_{(\mathit{o}\text{-HMBA})}$CT
				0.3909	HOMO→LUMO+11	M$_{(Au)}$L$_{(BINAP)}$CT mixed M$_{(Au)}$L$_{(\mathit{o}\text{-HMBA})}$CT
17	3.4341	361.03	0.0475	0.2778	HOMO-1→LUMO+4	M$_{(Au)}$L$_{(BINAP)}$CT mixed L$_{(\mathit{o}\text{-HMBA})}L_{(BINAP)}$CT
				0.5074	HOMO-1→LUMO+6	M$_{(Au)}$L$_{(BINAP)}$CT mixed L$_{(\mathit{o}\text{-HMBA})}L_{(BINAP)}$CT
				0.1968	HOMO→LUMO+9	M$_{(Au)}$L$_{(BINAP)}$CT
24	3.6035	344.07	0.0255	0.3333	HOMO-1→LUMO+5	M$_{(Au)}$L$_{(BINAP)}$CT mixed L$_{(\mathit{o}\text{-HMBA})}L_{(BINAP)}$CT
				0.4345	HOMO-1→LUMO+7	M$_{(Au)}$L$_{(BINAP)}$CT mixed L$_{(\mathit{o}\text{-HMBA})}L_{(BINAP)}$CT

32	3.7136	338.87	0.0320	0.2819	HOMO-8→LUMO	$L_{(o\text{-HMBA})}M_{(Au)}$CT mixed $L_{(o\text{-HMBA})}L_{(BINAP)}$CT
				0.2425	HOMO-7→LUMO	$L_{(o\text{-HMBA})}M_{(Au)}$CT mixed $L_{(BINAP)}M_{(Au)}$CT
				0.2526	HOMO-2→LUMO+1	$L_{(BINAP)}M_{(Au)}$CT
33	3.7354	331.91	0.0216	0.4022	HOMO-8→LUMO	$L_{(o\text{-HMBA})}M_{(Au)}$CT mixed $L_{(o\text{-HMBA})}L_{(BINAP)}$CT
36	3.7718	328.72	0.0362	0.2147	HOMO-2→LUMO+3	$L_{(BINAP)}L_{(BINAP)}$CT mixed $L_{(BINAP)}M_{(Au)}$CT
				0.4158	HOMO-2→LUMO+5	$L_{(BINAP)}L_{(BINAP)}$CT mixed $L_{(BINAP)}M_{(Au)}$CT
40	3.8135	325.21	0.0456	0.3299	HOMO-10→LUMO	$L_{(o\text{-HMBA})}L_{(BINAP)}$CT mixed $L_{(o\text{-HMBA})}M_{(Au)}$CT
				0.3127	HOMO-1→LUMO+9	$M_{(Au)}L_{(BINAP)}$CT mixed $L_{(o\text{-HMBA})}L_{(BINAP)}$CT
53	3.9430	316.05	0.0285	0.2718	HOMO-6→LUMO+2	$M_{(Au)}L_{(BINAP)}$CT mixed $L_{(o\text{-HMBA})}L_{(BINAP)}$CT
				0.2793	HOMO-7→LUMO+2	$M_{(Au)}L_{(BINAP)}$CT mixed $L_{(o\text{-HMBA})}L_{(BINAP)}$CT
64	4.0247	308.06	0.0122	0.2207	HOMO-12→LUMO	$L_{(BINAP)}M_{(Au)}$CT mixed $L_{(o\text{-HMBA})}L_{(BINAP)}$CT
67	4.0361	307.19	0.0467	0.26859	HOMO→LUMO+21	$M_{(Au)}L_{(BINAP)}$CT mixed $M_{(Au)}L_{(o\text{-HMBA})}$CT
				0.2885	HOMO-1→LUMO+13	$M_{(Au)}L_{(BINAP)}$CT
77	4.0846	303.54	0.0323	0.2200	HOMO-3→LUMO+6	$M_{(Au)}L_{(BINAP)}$CT
				0.2646	HOMO-2→LUMO+6	$L_{(BINAP)}M_{(Au)}$CT

Table S6. Frontier molecular orbital compositions (%) in the ground state for Au8c.

Orbital	Contribution (%)		
	Au core	S	P
HOMO-12	28.57	8.25	13.38
HOMO-10	29.64	20.50	3.49
HOMO-9	21.24	7.39	3.12
HOMO-8	31.47	12.98	3.26
HOMO-7	13.32	5.07	1.22
HOMO-6	21.84	18.67	4.39
HOMO-5	8.92	3.85	1.91
HOMO-4	5.88	0.85	1.60
HOMO-3	7.80	1.94	1.90
HOMO-2	4.65	1.97	1.16
HOMO-1	48.80	11.86	13.68
HOMO	67.79	4.15	13.69
LUMO	36.65	2.33	11.07
LUMO+1	12.26	1.41	5.32
LUMO+2	3.56	0.13	5.59
LUMO+3	10.32	0.35	8.35
LUMO+4	25.09	1.20	9.17
LUMO+5	17.36	1.11	7.92
LUMO+6	13.76	0.70	6.75
LUMO+7	27.81	1.24	12.25
LUMO+8	28.22	0.52	9.06
LUMO+9	16.83	0.51	13.82
LUMO+10	8.50	0.79	4.97
LUMO+13	12.77	1.32	10.95
LUMO+21	21.11	0.50	3.93

Table S7. Compositions (%) of different type shells in the ground state for Au8c.

Orbital	Contribution (%)			
	s	p	d	f
HOMO-12	8.58	71.97	19.42	0.024
HOMO-10	6.063	73.301	20.608	0.028
HOMO-9	8.609	78.613	12.768	0.010
HOMO-8	11.552	67.964	20.472	0.012
HOMO-7	4.857	86.695	8.442	0.005
HOMO-6	6.145	78.854	14.982	0.019
HOMO-5	3.099	91.931	4.962	0.008
HOMO-4	1.905	92.715	5.378	0.002
HOMO-3	3.305	91.448	5.241	0.005
HOMO-2	2.105	93.731	4.159	0.004
HOMO-1	15.942	57.242	26.785	0.031
HOMO	34.976	47.700	17.292	0.032
LUMO	14.341	80.410	5.230	0.019
LUMO+1	5.779	90.167	4.048	0.006
LUMO+2	4.211	92.057	3.729	0.003
LUMO+3	3.566	92.468	3.959	0.007
LUMO+4	11.457	83.244	5.286	0.012
LUMO+5	8.473	86.345	5.175	0.006
LUMO+6	8.139	87.301	4.549	0.010
LUMO+7	7.736	86.719	5.530	0.014
LUMO+8	5.859	90.190	3.937	0.013
LUMO+9	7.034	87.933	5.017	0.017
LUMO+10	5.150	91.315	3.530	0.004
LUMO+13	6.465	88.133	5.395	0.008
LUMO+21	5.336	91.108	3.548	0.008

Table S8. Results of CCM analyses of *R*-Au8 and *S*-Au8.

	***R*-Au8**		***S*-Au8**	
	**Structure**	**CCM**	**Structure**	**CCM**
**Core**	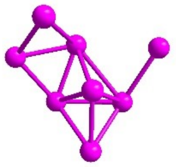	12.6045	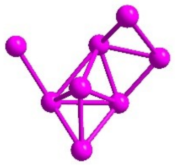	12.6045
**Core+Ligand**	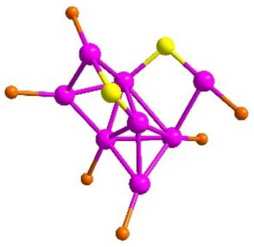	14.3970	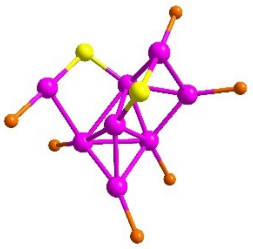	14.4242

Table S9. Hydrogen Bond and Angle Table for *R*-Au8c and S-Au8c.

***R*-Au8c**		
**O–H···O**	**Distance (Å)**	**Angle (°)**
O7–H7···O8	2.66(5)	159.8
O5–H5A···O9	2.79(5)	156.3
O4–H4A···O5	2.77(4)	144.7
O2–H2A···O3	2.65(3)	126.6
***S*-Au8c**		
**O–H···O**	**Distance (Å)**	**Angle (°)**
O9–H9A···O1	2.62(3)	171.3
O2–H2···O8	2.57(3)	130.1
O7–H7A···O8	2.78(4)	169.5
O3–H3A···O6	2.70(4)	164.5

Table S10. Crystal data and structure refinement for *R*-Au8d and *R*-Au8e.

Identification code	***R*-Au8d**	***R*-Au8e**
Empirical formula	$C_{146}H_{104}Au_8O_{10}P_6S_2$	$C_{146}H_{104}Au_8O_{10}P_6S_2$
Formula weight	3747.96	3747.96
Temperature/K	100	100
Crystal system	orthorhombic	orthorhombic
Space group	$P2_12_12_1$	$P2_12_12_1$
a/Å	20.5564(14)	20.333(5)
b/Å	25.7288(16)	25.812(5)
c/Å	26.0976(17)	26.048(5)
α/°	90	90
β/°	90	90
γ/°	90	90
Volume/Å^3	13802.8(16)	13671(5)
Z	4	4
ρ_{calc}g/cm^3	1.804	1.821
μ/mm^{-1}	16.890	17.053
F(000)	7064.0	7064.0
2Θ range for data collection/°	6.462 to 134.138	6.492 to 134.14
Index ranges	$-24 \le h \le 17$, $-30 \le k \le 30$, $-28 \le l \le 31$	$-22 \le h \le 24$, $-21 \le k \le 30$, $-27 \le l \le 31$
Radiation, CuKα	1.54184	1.54184
Reflections collected	80987	73670
Independent reflections	24480 [R_{int} = 0.0465, R_{sigma} = 0.0468]	23980 [R_{int} = 0.0601, R_{sigma} = 0.0615]
Data/restraints/parameters	24480/1050/1495	23980/426/1495
Goodness-of-fit on F^2	1.021	1.049
Final R indexes [I>=2σ (I)]	R_1 = 0.0457, wR_2 = 0.1107	R_1 = 0.0366, wR_2 = 0.0888
Final R indexes [all data]	R_1 = 0.0479, wR_2 = 0.1122	R_1 = 0.0403, wR_2 = 0.0903
Largest diff. peak/hole / e Å^{-3}	1.21/-1.90	1.36/-1.80
Flack parameter	0.041(4)	0.070(5)

Section 4. Reference

1. T. Hooper, C. Butts, M. Green, M. Haddow, J. McGrady, and C. Russell. *Chem-Eur J*, 2009, 15: 12196-12200.
2. Rigaku Oxford Diffraction. CrysAlis^{Pro} Software system, version 1.171.40.25a, Rigaku Corporation: Oxford, UK, 2018.
3. *APEX3, SAINT and SADABS*. Bruker AXS Inc., Madison, Wisconsin, USA, 2015.
4. L. Palatinus, and G. Chapuis. *J App Crystallogr,* 2007, 40: 786-790.
5. G. M. Sheldrick. *Acta Crystallogr Sect C,* 2015, 71: 3-8.
6. O.V. Dolomanov, L. J. Bourhis, R. J. Gildea, J. A. K. Howard, and H. Puschmann. *J Appl Crystallogr,* 2009, 42: 339-341.
7. A. L. Spek. *Acta Crystallogr Sect D,* 2009, 65: 148-155.
8. M. J. Frisch, G. W. Trucks, H. B. Schlegel, G. E. Scuseria, M. A. Robb, J. R. Cheeseman, G. Scalmani, V. Barone, G. A. Petersson, H. Nakatsuji, X. Li, M. Caricato, A. V. Marenich, J. Bloino, B. G. Janesko, R. Gomperts, B. Mennucci, H. P. Hratchian, J. V. Ortiz, A. F. Izmaylov and et al. Gaussian 16, Rev. B.01, Gaussian, Inc.: Wallingford, CT, 2016.
9. C. Adamo, and V. Barone. *J Chem Phys*, 1998, 108: 664-675.
10. S. Q. Li, B. Xu, Y. M. Xie, R. B. and King, H. F. Schaefer. *Dalton Trans*, 2007, 38: 4312-4322.
11. D.Y. Zubarev, and A.I. Boldyrev. *J Phys Chem A*, 2009, 113: 866-868.
12. T. Taketsugu, and A. Lyalin. *J Phys Chem Lett*, 2010, 1: 1752-1757.
13. T. Lu, and F. Chen. *J Comput Chem*, 2012, 33: 580-592.
14. Voss NR, Gerstein M. *Nucleic Acids Res*, 2010, 38: W555-W562.
